# Active learning for adaptive surrogate model improvement in high-dimensional problems

**DOI:** 10.1007/s00158-024-03816-9

**Published:** 2024-07-10

**Authors:** Yulin Guo, Paromita Nath, Sankaran Mahadevan, Paul Witherell

**Affiliations:** 1 Department of Civil and Environmental Engineering, Vanderbilt University, Nashville, TN 37235 USA; 2 Department of Mechanical Engineering, Rowan University, Glassboro, NJ 08028 USA; 3 Engineering Laboratory, National Institute of Standards and Technology, Gaithersburg, MD 20899 USA

**Keywords:** Surrogate model, Active learning, High dimension, Additive manufacturing

## Abstract

This paper investigates a novel approach to efficiently construct and improve surrogate models in problems with high-dimensional input and output. In this approach, the principal components and corresponding features of the high-dimensional output are first identified. For each feature, the active subspace technique is used to identify a corresponding low-dimensional subspace of the input domain; then a surrogate model is built for each feature in its corresponding active subspace. A low-dimensional adaptive learning strategy is proposed to identify training samples to improve the surrogate model. In contrast to existing adaptive learning methods that focus on a scalar output or a small number of outputs, this paper addresses adaptive learning with high-dimensional input and output, with a novel learning function that balances exploration and exploitation, i.e., considering unexplored regions and high-error regions, respectively. The adaptive learning is in terms of the active variables in the low-dimensional space, and the newly added training samples can be easily mapped back to the original space for running the expensive physics model. The proposed method is demonstrated for the numerical simulation of an additive manufacturing part, with a high-dimensional field output quantity of interest (residual stress) in the component that has spatial variability due to the stochastic nature of multiple input variables (including process variables and material properties). Various factors in the adaptive learning process are investigated, including the number of training samples, range and distribution of the adaptive training samples, contributions of various errors, and the importance of exploration versus exploitation in the learning function.

## Introduction

Engineering analyses such as reliability analysis, model calibration, and design optimization under uncertainty require multiple evaluations of the physics model (often for hundreds or thousands of realizations of the input). Detailed physics-based models usually have complicated geometry, material properties, loading, and boundary conditions, and finite-element-based models are often employed for large mechanical systems. It is expensive, and often affordable, to run these models a large number of times. Therefore, inexpensive surrogate models are constructed to replace the expensive, detailed physics-based models and efficiently map the model input to the output. In addition, for complex systems, the output quantities of interest (QoIs) can be multivariate outputs, which may be spatially or temporally correlated, and a function of a large set of variables in the input space. Therefore, surrogate modeling techniques need to be extended to problems with high-dimensional input and output.

The training data for the surrogate model is generated by multiple runs of the original physics model, and the accuracy of the surrogate model depends on the quantity and coverage of the training samples. For complex problems where the physics model is computationally expensive, generating a satisfactory number of training samples poses a significant challenge with respect to computational resources and time. Commonly used surrogate models, such as polynomial chaos expansion (PCE) (Xiu and Karniadakis 2002), Gaussian process (GP) surrogate models (Rasmussen and Williams 2006; Martin and Simpson 2005), support vector machines (SVM) (Cortes and Vapnik 1995) and neural networks (NN) (Haykin 2007) suffer from the curse of dimensionality in the presence of a large number of inputs and outputs (Guo et al. 2023).

Dimension reduction methods to overcome the challenge posed to surrogate modeling by high dimensionality have been reported in recent literature. For input dimension reduction, studies are reported on sparse grids (Ma and Zabaras 2009; Elsheikh et al. 2014); projection to an orthogonal basis, such as the truncated Karhunen-Loeve expansion (Marzouk and Najm 2009), proper orthogonal decomposition (POD) (Wang and Zabaras 2005), and combinations of both (Conrad and Marzouk 2013; Constantine et al. 2012; Winokur et al. 2013); and active subspace discovery in the input space (Constantine 2015; Zahm et al. 2020). For output dimension reduction, principal direction discovery has been implemented using principal components analysis (PCA) or singular vector decomposition (SVD) (Laub 2005; Hombal and Mahadevan 2013; Nath et al. 2017; Davis and Greenes 1983). For very high-dimensional outputs, PCA and SVD may become computationally unaffordable for high-dimensional problems due to the large size of the covariance matrix and limited computer memory in computers; in that case, randomized SVD (Halko et al. 2011) and random projection (Mahoney 2011) methods can be used to approximate the principal components and they are less computationally expensive compared to PCA and SVD. For problems with a large number of training samples, incremental PCA (Levy and Lindenbaum 1998; Ross et al. 2008) can overcome the memory limitations by processing the dataset in mini-batch fashion. In Giovanis and Shields (2020), a point-wise linear dimension reduction approach based on Grassmann manifold learning are used to construct surrogate models; in addition, nonlinear dimension reduction methods such as isomap (Balasubramanian and Schwartz 2002), locally linear embedding (Roweis and Saul 2000), Laplacian eigenmap (Belkin and Niyogi 2003), and diffusion map (Coifman et al. 2005; Coifman and Lafon 2006) aim to construct a representation of the data on a low-dimensional manifold embedded in a high-dimensional space and are known as manifold learning algorithms (Cayton 2005). Neural network-based autoencoder models have been studied for dimension reduction in image data (Wang et al. 2016) and anomaly detection (Kong and Mahadevan 2023). Recent work has considered both input and output dimension reduction, such as the principal component-active subspace (PCAS) method (Vohra et al. 2020; White et al. 2019), but is limited to tens of inputs and hundreds of outputs. Reference Guo et al. (2023) investigated a systematic way of comparing the different available strategies and finding the most suitable methods for surrogate modeling in high-dimensional problems with input dimension 27 and output dimension over 5000.

As stated above, another factor affecting the accuracy of the surrogate model is the coverage of the domain of interest by the training samples. It is crucial to develop an efficient design of experiments [DoE (Sacks et al. 1989)], i.e., the selection of the input values at which model simulations are conducted, to support accurate surrogate model construction with minimal computational cost. DoE methods can be classified as non-adaptive and adaptive. Non-adaptive DoE methods, or single-stage sampling strategies (Liu et al. 2018), determine the size and locations of the training samples in one single step. Rather than focusing on the physics of the input–output relationship, single-stage sampling methods focus on the coverage of the samples over the input space. Full-factorial (Dieter and Schmidt 2009), Latin hypercube (Stein 1987), orthogonal array (Owen 1992), minimax and maximin-distance designs (Johnson et al. 1990) are typically studied strategies in this category. Due to the lack of prior knowledge of the target function and limited computational resources, it is challenging to predetermine a proper size of the training samples; thus non-adaptive DoE methods could result in unsatisfactory surrogate models. By comparison, adaptive DoE methods use active learning-based sampling strategies that utilize information about the distribution of existing samples and the accuracy of the surrogate model built with the existing training samples; thus new samples can be sequentially added in regions not adequately covered by existing samples (this is referred to as exploration), or where the surrogate model accuracy is inadequate (this is referred to as exploitation). In addition, the adaptive process can stop when the desired accuracy is achieved by the surrogate model, thus saving computational resource and time. Compared to the non-adaptive methods, better performance of the adaptive methods has been observed in studies such as reliability analyses (Bichon et al. 2008; Zhou et al. 2019). Active learning strategies have also been developed for neural network models, such as for maximizing the information gain about the model parameters (MacKay 1992) or for minimizing the prediction errors (Cohn 1993). Reference Seo et al. (2000) demonstrates that they can also be applied to Gaussian Process regression models.

Methods to adaptively improve a surrogate model started with Jones’ expected improvement function (EIF) (Jones et al. 1998) in the context of optimization, which indicates how much the true value of the response at a point can be expected to improve on the current best solution. The expected feasibility function (EFF) (Bichon et al. 2008) extended this idea to reliability analysis, where the aim is to build a surrogate model that closely approximates a limit state, i.e., for a particular value (such as zero) of the input–output function. EIF and EFF are examples of learning functions; subsequent studies have proposed a variety of learning functions for adaptive improvement of the surrogate model, employed in Adaptive Kriging Monte Carlo simulation (AK-MCS) (Echard et al. 2011), combined importance sampling and adaptive Kriging (Echard et al. 2013; Dubourg et al. 2013), Kriging-based quasi-optimal importance sampling (Dubourg and Sudret 2014), and global sensitivity analysis-enhanced surrogate modeling (GSAS) (Hu and Mahadevan 2016). However, these methods are based on pure exploitation.

The learning function can also be formulated to further improve the sampling process by balancing exploration of the parameter space against exploitation of information about the performance of the surrogate model built from previously selected samples. Exploration involves selecting data points in unexplored regions of the design space, while exploitation suggests adding data points in regions where the surrogate model performed poorly. Exploitation approaches include gradient-based methods, committee-based methods, variance-based methods, bias-based methods, and cross-validation-based methods. Gradient-based adaptive sampling (Mackman and Allen 2010; Crombecq et al. 2011), uses the gradient or Hessian of the response to identify regions of prominent nonlinearity that are difficult to model. Committee-based methods (Douak et al. 2012; Hendrickx and Dhaene 2005) use a suite of different forms of surrogate models and select new points where the models disagree the most. Variance minimizing sampling (VMS) techniques (Seo et al. 2000; Tong 2001) identify where the model prediction has high uncertainty. Non-stationary processes can be represented using Gaussian trees and VMS algorithms have been proposed to optimize the design of samples (Gramacy and Lee 2008). Bias minimizing sampling (BMS) algorithms (Hombal and Mahadevan 2011) is an adaptive algorithm based on sequential realization of a hierarchical error decomposition model. The cross-validation (CV) approaches (Xu et al. 2014) divide a sample set into training and testing subsets to build and evaluate a model, respectively; additional samples are then placed in the region with the largest CV error, such as the accumulative leave-one-out (LOO) error approach (Li et al. 2010) which uses a weighted combination of LOO errors. Some geometry-based methods employ a combination of some of the methods mentioned above. The cross-validation Voronoi (CVVor) method (Xu et al. 2014) uses Voronoi tessellation to divide the input space into a set of Voronoi cells and uses a combination of cross-validation exploitation and distance-based exploration. The local linear approximation-Voronoi method (Crombecq et al. 2011) uses the gradient for exploitation and the volume of the Voronoi tessellation cells for exploration, to generate new samples in a discontinuous fashion. Active learning has also been used to sequentially partition the input random space and build localized PCE surrogate models (Novák et al. 2023).

However, the studies mentioned above have several limitations: (a) in some studies, adaptive improvement of the surrogate model is focused on a limit state (i.e., a particular value of the output, such as zero), rather than a function over the entire input space; (b) the focus is on a scalar output, with a small number of inputs; (c) high dimensionality of both the input and output spaces are not addressed simultaneously. Some recent studies have addressed one of these challenges, but not all of them. For example, reliability analysis considering high-dimensional output is addressed in Zhou and Peng (2021) and the adaptive surrogate modeling with only high-dimensional spatio-temporal output is addressed in Kapusuzoglu et al. (2022).

In this paper, we aim to address adaptive surrogate modeling for problems with high dimensionality in both the input and output spaces, in the presence of limited computational resources. Specifically, we first use SVD to map the output vector (size: thousands) to the principal component space and identify the dominant features. For each feature, we use the active subspace methodology to identify a low-dimensional subspace of the input domain and build a corresponding surrogate model. A new low-dimensional active learning strategy is proposed in this work to improve the surrogate models (one for each dominant feature) by adaptively adding new training samples using a learning function that balances exploration and exploitation. The exploration considers unexplored regions in the domain and the exploitation considers the high-bias regions of the domain. The proposed method is demonstrated for an additive manufacturing example, with a high-dimensional field output QoI, namely the residual stress in the manufactured part that has spatial variability due to the stochastic nature of multiple input variables, including process variables and material properties.

The methodology proposed in this paper has two novel features. First, compared to previous studies, in which the active learning is implemented in the original space and focuses on a scalar output, the proposed active learning strategy is conducted in a low-dimensional space. New samples are proposed in the low-dimensional space and can be easily mapped back to the original space; this is novel compared to previous studies in which the new training samples are directly generated in the original space, making them difficult to extend to high-dimensional applications. A second novel and critical feature of the methodology is that the active learning strategy is in coordination with dimension reduction in the output and input, which means that the active learning function should properly consider the mappings between different subspaces, since there is a different active subspace for each dominant output feature. Since multiple subspaces are considered, a new training sample proposed in one subspace has different coordinates and effects in the other subspaces; this interaction is properly accounted for in the proposed methodology. The proposed learning function can improve multiple surrogate models simultaneously.

An additional contribution of this paper is that several factors in the adaptive learning process (in coordination with various space mappings) are investigated, such as the number of training samples in each iteration, range and distribution of the candidate samples for adaptive learning, the weighting of exploration vs. exploitation in the learning function, and contributions of different error sources. These investigations directly relate to the aspects mentioned in the preceding paragraph, and provide insights and guidance to applying the proposed method to practical engineering problems.

The rest of the paper is organized as follows. In Sect. 2, the method for surrogate model construction with high-dimensional input and output is first presented, followed by the proposed method for adaptive improvement of the surrogate model, with a focus on the active learning function and the issues affecting improvement. The proposed methodology is then demonstrated using three benchmark test functions in Sect. 3. The numerical example related to additive manufacturing is presented in Sect. 4 and the proposed methodology is evaluated by investigating the various factors mentioned above. Section 5 provides concluding remarks, summarizes the contributions of this paper, and identifies future research needs.

## Methodology

The proposed methodology aims to efficiently construct surrogate models for problems with high-dimensional input and output with a limited number of possible training runs of the original physics model, by pursuing an active learning strategy to adaptively select the training runs. In this section, we first introduce the dimensional reduction techniques in both input and output spaces. The surrogate is built using the lower-dimensional representation of the original high-dimensional output (referred to as ‘features’ in this work) as the output, and the corresponding inputs in the active subspace as the inputs. Then, the adaptive improvement process with a learning function is developed, and issues affecting the improvement are discussed.

### Output dimension reduction

Physics-based computational modeling, such as finite element analysis, of a large structure provides many output quantities, such as stresses and deformations corresponding to all degrees of freedom, at all locations on the structure. It is not practical to directly build individual, separate surrogate models for every output at every location, although it is possible when some assumptions about the input correlations are made [for example, if the same correlation structure is assumed throughout the domain for the outputs at all the spatial locations (Gu and Berger 2016)]. Moreover, the multiple outputs are correlated as they share common inputs; therefore, the correlations need to be accounted for when building the surrogate models for multiple outputs. Co-Kriging (Han et al. 2012) can be used to build surrogate models for correlated outputs but it has been demonstrated only for a small number of dimensions. A more practical option is to map the high-dimensional output to an uncorrelated space and then build individual surrogate models in the uncorrelated space.

In Guo et al. (2023), a systematic approach for identifying the most suitable dimension reduction techniques for a given problem was introduced. Based on the size of the output, computational resources, and desired accuracy, techniques including SVD, random projection, randomized SVD, and diffusion map could be utilized for output dimension reduction. In this paper, we use SVD for reducing the output dimension for the sake of illustration. We briefly introduce SVD here; for any other problem, the best dimension reduction technique could be selected using the approach referenced above.

SVD is a generalized eigen-decomposition dimension reduction method by projecting the original data along the first few orthogonal principal directions that capture most of the variance in the data. The SVD method maps the correlated variables to an uncorrelated space. Note that it will not work for variables that are uncorrelated.

For ${\varvec{X}} \in {\mathbb {R}}^{M \times N}$, SVD is the factorization ${\varvec{X}}={\varvec{U}} \varvec{\Sigma } {\varvec{V}}^{\prime }=\sum _{k=1}^{\min (M, N)} \sigma _{k} {\varvec{u}}_{k} {\varvec{v}}_{k}^{\prime }$, where ${\varvec{U}} \in {\mathbb {R}}^{M \times M}$ and ${\varvec{V}} \in {\mathbb {R}}^{N \times N}$ are unitary matrices that consist of left singular vectors ${\varvec{u}}_{k}$ and right singular vector ${\varvec{v}}_{k}$, respectively. Diagonal matrix $\varvec{\Sigma } \in {\mathbb {R}}^{M \times N}$ contains singular values $\sigma _1, \sigma _2, \ldots , \sigma _p$, $p = min\{M,\ N\}$ and $\sigma _1 \geqslant \sigma _2 \geqslant \ldots \geqslant \sigma _p$. The amount of variance explained by the *i*-th singular value and its corresponding singular vectors ${\varvec{u}}_{i}$ and ${\varvec{v}}_{i}$ is given by $R^2_{SVD} = \sigma _i^2/\Sigma _j \sigma _j^2$. With the top *k* largest singular values in $\varvec{\Sigma }$ and the corresponding first *k* columns from ${\varvec{V}}$, an approximation of the original matrix ${\varvec{X}}$ can be reconstructed by $\hat{{\varvec{X}}} = {\varvec{U}}_k\varvec{\Sigma }_k{\varvec{V}}_k'$, where ${\varvec{U}}_k$ is a matrix containing the first *k* left singular vector, $\varvec{\Sigma }_k$ is the first *k* singular values organized in a $k\times k$ diagonal matrix and ${\varvec{V}}_k$ is a matrix containing the firs *k* right singular vectors.

A lower-dimensional representation (dimension *r*) in place of the original data (dimension *N*) can be taken as

1 $$\begin{aligned} \hat{{\varvec{X}}}^{LD} = {\varvec{U}}_k\varvec{\Sigma }_k, \end{aligned},$$

where $\hat{{\varvec{X}}}^{LD} \in {\mathbb {R}}^{M \times k}$ contains *M* *k*-dimensional points. These points can be viewed as the coordinates on the orthonormal basis $[{\varvec{v}}_1, {\varvec{v}}_2, \ldots , {\varvec{v}}_k]$. We will refer to these coordinates as ‘features’. These features are uncorrelated by definition but not independent. Note that due to the orthonormality of right singular vectors ${\varvec{v}}_i$’s, the mapping between the lower-dimensional representation $\hat{{\varvec{X}}}^{LD}$ and the higher-dimensional value ${\varvec{X}}$ is exact with the knowledge of ${\varvec{V}}_k$: $\hat{{\varvec{X}}} = \hat{{\varvec{X}}}^{LD}{\varvec{V}}_k$.

It is important to note that dimension reduction via SVD is a linear transformation, which may not work for highly nonlinear data. (In that case, nonlinear dimension reduction methods mentioned earlier, such as diffusion maps or autoencoder, could be used.) The SVD algorithm also involves the calculation of the correlation matrix of the original data matrix and requires more computational resources for a large dataset.

Consider a spatially varying field quantity as the output, i.e., ${\varvec{S}} = {\varvec{S}}(\varvec{\theta })$, where $\varvec{\theta } \in \varvec{\Omega }$ is the input on its domain $\varvec{\Omega }$. The field being considered ${\varvec{S}}(\varvec{\theta })\ \in {\mathbb {R}}^{r\times c}$ is evaluated on a two-dimensional mesh of size $(r\times c)$ for a given set of inputs $\varvec{\theta }$ and ${\varvec{S}}(\varvec{\theta })$ is available at $N_s$ pseudorandom samples, drawn from the joint probability density function (PDF) of $\varvec{\theta }$. A data matrix ${\varvec{X}} \in {\mathbb {R}}^{N_s \times (r\times c)}$ is first constructed using the field data at $N_s$ samples, each row of ${\varvec{X}}$ contains the matrix ${\varvec{S}}$, reshaped as a row vector of size $(r\times c)$.

As mentioned earlier, we refer to the low-dimensional representation of the high-dimensional output as features. Suppose we use a matrix ${\mathscr {L}}$ to denote these features. ${\mathscr {L}}$ has $N_s$ rows, each containing feature values corresponding to a sample. The number of columns in ${\mathscr {L}}$ can be determined using the amount of explained variance for the specific engineering problem, i.e., equal to the number of singular vector-singular value pairs $(K^*)$ that is sufficient for reconstructing the field ${\varvec{S}}$ with desired accuracy. The feature matrix ${\mathscr {L}}$ can be mathematically represented as:

2 $$\begin{aligned} {\mathscr {L}}=\left[ \begin{array}{cccc} {\mathscr {L}}_{11} &{} {\mathscr {L}}_{21} &{} \cdots &{} {\mathscr {L}}_{K^* 1} \\ {\mathscr {L}}_{12} &{} {\mathscr {L}}_{22} &{} \cdots &{} {\mathscr {L}}_{K^* 2} \\ \vdots &{} \vdots &{} \ddots &{} \vdots \\ {\mathscr {L}}_{1 N_s} &{} {\mathscr {L}}_{2 N_s} &{} \cdots &{} {\mathscr {L}}_{K^* N_s} \end{array}\right] \end{aligned}$$

Correspondingly, $\hat{{\varvec{S}}}$ (the approximation of ${\varvec{S}}$) will be each row of the product of the matrices, ${\mathscr {L}}$ and ${\varvec{V}}_{K^*}$, reshaped to a matrix of dimension *r*-by-*s*. Note that each element ${\mathscr {L}}_{ji}$ in matrix ${\mathscr {L}}$ represents a value of a particular feature *j* corresponding to a sample *i*. This is an abstract value in the low-dimensional latent space; one such value does not represent any physical value (output in the original high-dimensional space). Each element $X_{im}$ in ${\varvec{X}}$ represents a physical QoI in the physical space at a particular point *m* in the mesh of size $r\times c$, corresponding to the sample *i* of a total size $N_s$.

### Input dimension reduction

In contrast to dimension reduction by sensitivity analysis, where insignificant variables are dropped or treated as a constant using their nominal values while dominant variables are kept for further analysis, the active subspace approach simply maps all the original inputs to a lower-dimensional space without dropping any variables; the mapped inputs are linear combinations of the original inputs. An active subspace is a low-dimensional subspace that consists of important directions in a model’s input space (Constantine 2015); that is, most of the variability in a model output *f* due to the uncertain inputs is captured along these important directions. The directions constituting the active subspace are the dominant eigenvectors of the uncentered covariance matrix ${\varvec{C}}=\int _{\Omega }\left( \nabla _{\varvec{\xi }} f\right) \left( \nabla _{\varvec{\xi }} f\right) ^{\top } \mu (d \varvec{\xi })$, which is a positive semidefinite matrix with $\mu (d \varvec{\xi }) = \pi (\varvec{\xi })d (\varvec{\xi })$, where $\pi (\varvec{\xi })$ is the joint probability density function of $\varvec{\xi }$. The random vector $\varvec{\xi } \in \Omega \in {\mathbb {R}}^{N_{p}}$ is the vector of uncertain model inputs, $N_p$ is the number of the uncertain inputs; *f* is assumed to be a square integrable function with continuous partial derivatives of the output with respect to the inputs; the partial derivatives are further assumed to be square integrable. $\nabla _{\varvec{\xi }} f$ is the gradient vector of *f* with respect to $\varvec{\xi }$. Since ${\varvec{C}}$ is symmetric and positive semidefinite, it can be decomposed as ${\varvec{C}}={\varvec{W}}\varvec{\Lambda }{\varvec{W}}^{\top }$, where $\varvec{\Lambda }=diag(\lambda _1, \ldots , \lambda _{N_{p}})$ with the eigenvalues $\lambda _i$’s sorted in descending order $\lambda _{1} \geqslant \lambda _{2} \geqslant \ldots \geqslant \lambda _{N_{\textrm{p}}} \geqslant 0$, and ${\varvec{W}}$ has the (orthonormal) eigenvectors ${\varvec{w}}_1,\ \ldots , {\varvec{w}}_{N_{p}}$ as its columns. The eigenpairs are partitioned about the *r*th eigenvalue such that $\lambda _r/\lambda _{r+1} \gg 1$,

3 $$\begin{aligned} {\varvec{W}}=\left[ {\varvec{W}}_{1}\ {\varvec{W}}_{2}\right] , \quad {\varvec{\Lambda }}=\left[ \begin{array}{cc} {\varvec{\Lambda }}_{1} &{} \\ &{} {\varvec{\Lambda }}_{2} \end{array}\right] \end{aligned}$$

The columns of $\varvec{W_1} = [{\varvec{w}}_1 \dots {\varvec{w}}_r]$ span the dominant eigenspace of ${\varvec{C}}$ and define the active subspace, and ${\varvec{\Lambda }}_{\textbf{1}}$ is a diagonal matrix with the corresponding set of eigenvalues, $\lambda _1, \ldots , \lambda _r$, on its diagonal. Once the active subspace is computed, dimension reduction is accomplished by transforming the parameter vector ${\varvec{\xi }}$ into ${\varvec{y}} = {\varvec{W}}_1^{\top } \varvec{\xi } \in {\mathbb {R}}^r$.

The active subspace discovery algorithm starts with the information about the gradients of the output with respect to the input variables. A regression-based approach can be used to estimate the gradient of *f* in a global sense using a linear regression-fit to the available samples from a set of model evaluations (Vohra and Mahadevan 2019), in which the number of model evaluations can be increased iteratively based on the convergence of the dominant eigenvectors of ${\varvec{C}}$. This approach is adapted from Algorithms 1 and 2 in Constantine (2015) and enhances their efficiency using an iterative approach, and the details can be found in Vohra and Mahadevan (2019). The core idea of this approach is to first estimate the gradient of the model output, *f*, and hence the matrix ${\varvec{C}}$, whose eigenvalues can be found using more samples iteratively. Suppose a set of model input–output pairs $\{(\varvec{\xi }_p, f_p)\}_{i = 1}^{N_p}$ is available for estimating the active subspace, an independent set of samples of input $\{\varvec{\xi }_j\}_{j = 1}^{N_j}$ is first drawn from $\pi (\varvec{\xi })$ using Monte Carlo sampling. Note that this set of input settings are used for finding *s* samples in $\{(\varvec{\xi }_p, f_p)\}_{i = 1}^{N_p}$ in a bootstrapping manner, therefore, model evaluations for these $N_j$ input settings are not necessary. For each sample (input setting only) in $\{\xi _j\}_{j = 1}^{N_j}$, a least-squares fit to *s* nearest neighbors in $\{(\varvec{\xi }_p, f_p)\}_{i = 1}^{N_p}$ is performed and the slope vector ${\varvec{d}}_j$ of the linear regression-fit is recorded. Note that the choice of *s* can range between 2 and $N_p - 1$; $s = N_p - 1$ captures the gradients in a leave-one-out manner. Based on the slope determined by the least-square fit using *s* samples in $\{(\varvec{\xi }_p, f_p)\}_{i = 1}^{N_p}$ corresponding to each input setting in $\{\varvec{\xi }_j\}_{j = 1}^{N_j}$, ${\varvec{C}}$ can be estimated as $\varvec{\hat{C}} \approx \frac{1}{N_j}\Sigma _{j=1}^{N_j} {\varvec{d}}_j {\varvec{d}}_j^{\top }$. By performing eigenvalue decomposition of $\varvec{\hat{C}}$, the active subspace can be estimated as described in Eq. (3). Note that the estimation of the subspace can start with $N_p$ samples, and more samples can be added iteratively. At each iteration, an active subspace, whose mapping can be estimated by ${\varvec{W}}_1$, can be estimated. This iterative process stops when the difference between estimations of ${\varvec{W}}_1$ in two consecutive iterations are smaller than a pre-determined threshold. The active subspace technique works best when there exists a trend between the output and the input, as indicated by the construction of ${\varvec{C}}$ shown above. If there is no clear trend for the output in terms of the input, this technique will not be effective in reducing the dimension of the surrogate model input.

In this work, we are interested in the relationship between each feature, i.e., each column of matrix ${\mathscr {L}}$, $[{\mathscr {L}}_1, {\mathscr {L}}_2, \ldots , {\mathscr {L}}_j, \ldots , {\mathscr {L}}_{K^*}]$ and the input variables $\varvec{\theta }$. The *j*-th feature, ${\mathscr {L}}_j$, contains $N_s$ realizations of feature *j* in the low-dimensional space capturing the variability in feature *j* due to variability in $N_s$ input values of $\varvec{\theta }$. ${\mathscr {L}}_j$ can then be treated as a scalar-valued function of the input $\varvec{\theta }$, ${\mathscr {L}}_j(\varvec{\theta })$. An active subspace can now be defined as a low-dimensional subspace in the input domain that effectively captures the variability in ${\mathscr {L}}_j$ due to variations in $\varvec{\theta } \in {\mathbb {R}}^{N_\theta \times 1}$. $N_{\theta }$ denotes the number of uncertain inputs. The active subspace is spanned by the dominant eigenvectors of the uncentered covariance matrix containing the derivative information of ${\mathscr {L}}_j$ with respect to the components of $\varvec{\theta }$. Correspondingly, the active variables can be calculated as $\varvec{\eta } = {\varvec{W}}_1^{\top } \varvec{\theta }$, $\varvec{\eta } \in {\mathbb {R}} ^ {N_{\eta } \times 1}$, where $N_{\eta }$ is equal to the number of eigen vectors in ${\varvec{W}}_1$ of the uncentered covariance matrix. Input dimension reduction is achieved when $N_{\eta } < N_{\theta }$.

### Surrogate modeling in the active subspace

As discussed above, dimension reduction is first performed for the high-dimensional field output QoI, to select a small number of independent variables, i.e., dominant features in matrix ${\mathscr {L}}$. The input dimension reduction is subsequently considered, resulting in an active variable vector $\varvec{\eta }$ that has a smaller dimension than the original input vector $\varvec{\theta }$. Note that each feature ${\mathscr {L}}_j$ has a different active variable vector $\varvec{\eta }$. We define the functional relationship between the dominant features (representing the output) ${\mathscr {L}}$ and the corresponding active variables (representing the input) $\varvec{\eta }$ as $G_j(\varvec{\eta })$. Note that this function is only implicitly known to us in the sense that we only have $N_s$ samples. Then the dependence of each individual feature on its corresponding active variables can be approximated using a surrogate model $\hat{G}_j(\varvec{\eta })$ in the active subspace. For a low-dimensional surrogate model (i.e., one or two inputs), a polynomial regression-fit may sometimes be an adequate choice; otherwise, PCE, GP or other sophisticated surrogate models could be chosen. In summary, an active subspace is calculated for each dominant feature ${\mathscr {L}}_j$ and a surrogate model $\hat{G}_j$ is constructed in the active subspace for $j = 1, 2, \ldots , K^*$. This process is shown in the following algorithm.

**Algorithm 1:**
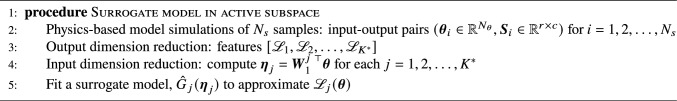
Surrogate model in active subspace

Note that (1) the $K^*$ dominant features are uncorrelated with each other, and separate surrogate models are built for each feature; (2) the active subspaces corresponding to different features ${\mathscr {L}}_j$ for $j = 1, 2, \ldots , K^*$ are different. The separate surrogate models for all the dominant features will be used together to reconstruct the approximated high-dimensional output in the original space.

### Proposed active learning strategy for adaptive surrogate improvement

If the physics model is computationally expensive, it may be unaffordable to generate a large number of training samples for the surrogate model when limited computational resources are available. With a small number of initial training samples, the constructed surrogate model might be quite approximate and might need to be improved to achieve the desired accuracy. Three key components are considered in the proposed active learning strategy for adaptive improvement: (a) the initial training samples, (b) the learning function to help select new training samples, and (c) the stopping criterion. In the proposed strategy, two sets of samples are first generated. One set of samples is used to build the initial surrogate model, and the other is used as a testing set. An active learning function that combines exploration and exploitation is proposed, in order to choose new training samples that are added to the first set for updating the surrogate model in each iteration; the testing set will remain unchanged throughout the adaptive improvement process. The stopping criterion is chosen based on the specific engineering application and the accuracy requirement.

When the input is high-dimensional, a grid of uniformly spaced samples along every dimension may result in a large number of initial samples, and running the original expensive physics model for all those samples may be unaffordable with limited computational resources. Therefore the initial set of training samples in the proposed approach is generated using an optimal symmetric Latin hypercube (OSLH) sampling technique. This technique helps to achieve the best coverage of the input domain for any given number of samples. The testing sample set is also generated separately using OSLH. We denote the initial training samples as ${\mathscr {D}}_0 = \{(\varvec{\theta }_i, {\varvec{S}}_i)\}_{i = 1}^{N_0}$, and the testing sample set as ${\mathscr {D}}_t = \{(\varvec{\theta }_i, {\varvec{S}}_i)\}_{i = 1}^{N_t}$. The initial surrogate model is constructed with ${\mathscr {D}}_0$.

The active learning function is then employed to iteratively add new training samples to ${\mathscr {D}}_0$ in order to adaptively improve the surrogate model. One straightforward way is to generate new training points in the neighborhood where the surrogate has a high prediction error. However, such a strategy is purely based on exploitation, which can lead to clustered samples in the input space. The surrogate model is desired to accurately approximate the field quantity of interest ${\varvec{S}}$ for any given $\varvec{\theta }$; such clustering of samples will not efficiently improve the surrogate model over the entire domain. Although all the dominant features’ surrogates contribute to the estimation the high-dimensional output ${\varvec{S}}$ in the original space, their contributions are not equally important; this must also be taken into consideration in constructing the learning function.

In order to address these two issues, a desirable active learning function should have two properties: (1) be able to balance exploration and exploitation; and (2) consider the differences in the contributions of the dominant features to the output ${\varvec{S}}$. Both exploration and exploitation are important in finding new training samples to improve the surrogate model. In this paper, exploration is defined as finding new samples in unexplored region of the input domain, exploitation is defined as finding new samples in regions where the surrogate model performs poorly. A learning function in terms of the active variables $\varvec{\eta }$ corresponding to the most dominant feature is thus proposed:

4 $$\begin{aligned} {\mathcalligra{l}}(\varvec{\eta })= & {} \alpha \times \left( \frac{w_1\sum _{j=1}^N\Vert \varvec{\eta }-\varvec{\eta }_j^{\text {train}, 1}\Vert }{N \cdot \max _{p \ne q} \Vert \varvec{\eta }_p^{\text {train}, 1} - \varvec{\eta }_q^{\text {train}, 1}\Vert }\nonumber \right. \\{} & {} \left. +\sum _i \frac{w_i \sum _{j=1}^N\Vert \varvec{\eta }_{i}-\varvec{\eta }_{j}^{\text {train}, i}\Vert }{N \cdot \max _{p \ne q} \Vert \varvec{\eta }_p^{\text {train}, i} - \varvec{\eta }_q^{\text {train}, i}\Vert }\right) \nonumber \\{} & {} +(1-\alpha ) \times \delta \left( \varvec{\eta }\right) \end{aligned},$$

where $\varvec{\eta }$ is the new candidate training point in the active subspace of the most dominant feature (feature 1), and is the optimization variable in the learning function. This learning function has two basic terms, exploration term with a weight $\alpha$ and exploitation term with a weight $(1-\alpha )$. The weighting factor $\alpha \in [0, 1]$ balances exploration and exploitation. The exploration term uses a distance-based metric to explore unsampled regions in the domain whereas the exploitation term aims to identify regions with large prediction error. Within the exploration term, the various dominant features are weighted based on how much of the variance in the output is captured by each feature, i.e., $w_i = \sigma _i^2\ / (\Sigma _{m=1}^{K*}\sigma _m^2)$, where $m = 1, 2, 3, \ldots , K*$, and $K*$ is the number of dominant features. In the above equation, *i*=1 for the first exploration term corresponding to feature 1, and *i* = 2, 3, ..., $K*$ for the second term corresponding to the other dominant features.

The goal of exploration is to find the least-sampled region of the domain given the existing training samples, but note that all the dominant features and their subspaces need to be considered. We formulate the exploration part with two terms: a single ratio and a sum of ratios. All ratios are multiplied by a weighting factor $w_i$. The reason for the two terms is that the new training sample is chosen in the active subspace of the first dominant feature, and then mapped to the active subspaces corresponding to other features.

The first term in the exploration part considers the first dominant feature. The numerator quantifies the Euclidean distance between the new candidate training point $\varvec{\eta }$ and the previous *N* training samples $\{\varvec{\eta }_j^{\text {train}, 1}\}_{j = 1}^{N}$. The denominator serves as a normalizing factor, which is *N* times the maximum largest pairwise Euclidean distance among all *N* previous training points.

**Fig. 1:**
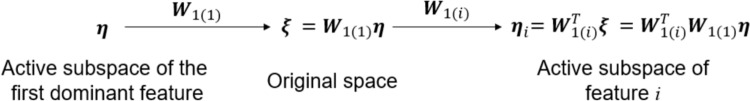
Projection from the active subspace of the first dominant feature to active subspaces of other features

The second term in the exploration part contains the ratios of all other dominant features other than feature 1; since the active subspace associated with each feature is different, the new candidate training point $\varvec{\eta }$ in the active subspace of the first dominant feature is mapped to the active subspace of the corresponding feature *i* using the matrices ${\varvec{W}}_{1(1)}$ and ${\varvec{W}}_{1(i)}$ and their orthonormal properties, $\varvec{\eta }_{i} = {\varvec{W}}_{1(i)}^{\top }{\varvec{W}}_{1(1)}\cdot \varvec{\eta }$. This projection is illustrated in Fig. 1. Note that if the active subspace mapping for a non-focus feature is orthogonal to that of the focus feature, i.e., ${\varvec{W}}_{1(1)}^{\top }{\varvec{W}}_{1(i)} = 0$ for $i \ne 1$, then the contribution from feature *i* becomes zero in the exploration term of the learning function. As a result, the surrogate model for non-focus feature *i* may not be improved using the proposed learning function, which is a function of the active variable corresponding to the focus feature. However, in this case, if the variance explained by the focus feature is significantly larger than that of the non-focus feature, the learning function will still serve the purpose. Also, although the features themselves are orthogonal to each other, the corresponding active subspace mapping vectors (${\varvec{W}}_{1(1)}$, ${\varvec{W}}_{1(2)}$,..., ${\varvec{W}}_{1(i)}$) need not be orthogonal to each other. Nonetheless, one should check ${\varvec{W}}_{1(1)}^{\top }{\varvec{W}}_{1(i)}$ during the learning process. Also note that the dimension of $\varvec{\eta }$ could be different from that of $\varvec{\eta }_{i}$, since different features have different active subspaces (defined by ${\varvec{W}}_{1(1)}$ and ${\varvec{W}}_{1(i)}$ as shown in Sect. 2.2) and the subspaces could have different dimensions.

By calculating the sum of distances and normalization, the region that is the furthest away from all available training samples is identified, thus the least-sampled region can be explored. Different active subspaces corresponding to the first dominant feature and other dominant features are incorporated in the calculation through proper mapping. In addition, the dominant features are weighted based on the output variance captured by each feature, as explained above.

The goal of exploitation is to find regions with high surrogate model error. For any new candidate training sample, it is unaffordable to run the expensive computational physics model and estimate the surrogate error; therefore alternative methods to estimate the surrogate error need be pursued. Such options include building additional (secondary) surrogate models to estimate the error based on the results of the previous training points (Nath et al. 2019), or simply using the surrogate error at the existing training sample that is closest to the candidate training sample. Mathematically, with the second option, the exploitation term can be formulated as $\delta \left( \varvec{\eta }\right) = RMSE(\hat{{\varvec{S}}}(\varvec{\eta }_{nr}))/\max \{ RMSE(\hat{{\varvec{S}}}(\varvec{\eta }^{\text {train}}))\}$, where $\varvec{\eta }_{nr}$ is the training sample with the smallest Euclidean distance to the candidate sample $\varvec{\eta }$ (nearest neighbor and thus the subscript), $\hat{{\varvec{S}}}(\cdot )$ is the original space QoI prediction, and RMSE is the root mean square error. The denominator is a normalizing factor that ensures the value of the exploitation part to be within the range of [0, 1], which is also the case for the exploration part. The contributions of all features can be considered in the exploitation term. The term $\delta (\varvec{\eta })$ can be based on metrics in the original space, like normalized RMSE of the QoIs. Since the original space QoIs are obtained using all dominant features, one single metric includes contributions of all features. Although this error estimation is approximate, it is only for the sake of new sample selection. As the adaptive learning proceeds with more new samples in more iterations, this error is expected to be reduced. Also note that the exploitation term is based on the bias of the surrogate model because it is shown that using the bias captures nonlinearities in the underlying function more effectively than using the variance as the criterion (Hombal and Mahadevan 2011).

In summary, the learning function considers all significant PCs and their corresponding active variables. The exploration term assigns a relative weight to each significant PC based on the variance captured by each PC, and the exploitation term considers the contribution of each significant PC to the error in the QoI in the original space by means of the mapping to the original space. As a result, the proposed new training sample balances the contributions of all significant PCs.

Overall, the learning function consists of two sets of weighting factors. The feature weighting factors within the exploration term are determined by the problem, whereas the factor $\alpha$ that balances exploration and exploitation is chosen subjectively by the analyst.

The new training sample $\varvec{\theta }^*$ can be obtained in two steps: (1) find the maximizer of the learning function $\varvec{\eta }^* = argmax({\mathcalligra{l}}(\varvec{\eta }))$; (2) transfer $\varvec{\eta }^*$ back to the original input domain $\Omega$, $\varvec{\theta }^* = {\varvec{W}}_{1(1)}^{\top }\varvec{\eta }^*$, by taking advantage of the orthonormal property of the basis vectors that span the active space associated with the first dominant feature. In order to find the maximizer of the learning function ${\mathcalligra{l}}(\varvec{\eta })$, sampling-based optimization is adopted here considering the challenge posed by the complicated learning function that is constructed based on distance calculations between samples (in different spaces). A candidate pool ${\mathscr {P}}_{\eta }$ is first generated based on the information provided by the initial surrogate models constructed with ${\mathscr {D}}_0$. The learning function ${\mathcalligra{l}}(\cdot )$ is then evaluated at each sample within ${\mathscr {P}}_{\eta }$ to select the maximizer $\varvec{\eta }^*$. This can be expressed as:

$$\begin{aligned} \begin{aligned}&\underset{\varvec{\eta }}{\text {argmax}}{} & {} {\mathcalligra{l}}(\varvec{\eta }) \\&\text {subject to}{} & {} \varvec{\eta } \in {\mathscr {P}}_{\eta } \end{aligned} \end{aligned}$$

The physics-based model can then be evaluated at the corresponding $\varvec{\theta }^*$ to obtain ${\varvec{S}}^*$ and the dataset of training samples is updated as ${\mathscr {D}}_i = {\mathscr {D}}_{i - 1} \bigcup \{(\varvec{\theta }^*, {\varvec{S}}^*)\}$, where *i* indicates the iteration number. Note that all $\varvec{\eta }^*$’s are selected from ${\mathscr {P}}_{\eta }$ without replacement.

The iterative selection of training samples ends when the predefined stopping criterion (e.g., the desired accuracy of the surrogate prediction) is achieved.

Note that we have three sets of data in the workflow. Two sets contain input–output pairs, a training set (${\mathscr {D}}_i$, where *i* is the iteration number, *i* = 0, 1, 2, $\ldots$) and a testing set (${\mathscr {D}}_t$). One set only contains input settings, the candidate pool, ${\mathscr {P}}$, for choosing additional samples; the candidates in this pool are not input–output pairs like ${\mathscr {D}}_i$ or ${\mathscr {D}}_t$. Rather, the candidate samples are in the low-dimensional active variable space; once one of the candidate samples is chosen, it will be mapped back to the original high-dimensional space and evaluated in the detailed physics model to obtain the corresponding output. Note that the surrogate model is constructed using the training set ${\mathscr {D}}_i$, and evaluated on the testing set ${\mathscr {D}}_t$. In each iteration, a new sample is selected from the candidate pool ${\mathscr {P}}$ using the learning function. Note that as shown in Hombal and Mahadevan (2011), variance-based sampling gives a uniform distribution of samples whereas bias minimization gives samples in high-bias regions. Thus prediction uncertainty alone is not enough and requires more samples than bias minimization. And bias estimation requires a test set.

The selection of the new sample is not based on error on the testing set, but it is based on the values of the learning function for the candidate samples in the active variable space; the candidate sample with the maximum value of the learning function is chosen. The learning function has two components, exploration and exploitation. The former calculates a metric based on the distance of a proposed sample to all existing samples in ${\mathscr {D}}_i$, and the latter calculates the error of the surrogate prediction at a proposed sample. In the benchmark test functions, the surrogate error can be directly calculated, since it is not expensive to evaluate the original function in Sect. 3; in the additive manufacturing example in Sect. 4, the error is approximated by that of the closest existing training sample in ${\mathscr {D}}_i$ to a proposed sample.

Note that the active learning strategy in the low-dimensional space is independent of the dimension reduction method, which simply provides a mapping between the original space and the low-dimensional space. Linear dimension reduction methods are presented in this paper for demonstration purposes. Nonlinear dimension reduction methods, such as isomap (Balasubramanian and Schwartz 2002), locally linear embedding (Roweis and Saul 2000), Laplacian eigenmap (Belkin and Niyogi 2003), and diffusion map (Coifman et al. 2005; Coifman and Lafon 2006), can also be used depending on the specific application. Any dimension reduction method will contribute to the total prediction error; the error due to dimension reduction can be quantified as discussed in Guo et al. (2023).

### Issues affecting adaptive improvement

There are several issues that affect the active learning process, as follows:

1. In the active learning process, new training samples are added at each iteration, so the total number of samples in each iteration is different from that of the previous iteration. In any iteration *i* (where there are $N_i$ samples for surrogate model construction), there are two different options for space mapping calculations. The first option is to always project to the initial feature space and the corresponding active subspaces, using ${\varvec{V}}^{(0)}$ and ${\varvec{W}}^{(0)}$ calculated by the initial $N_0$ samples. The second option is to recalculate both the feature and active spaces with the available $N_i$ samples at the *i*-th iteration, and the corresponding projections are denoted as ${\varvec{V}}^{(i)}$ and ${\varvec{W}}^{(i)}$. Different options will result in different feature and active variable values, which may affect the adaptive improvement of the surrogate model. Using option 1 in all iterations implies that the initial subspaces can represent the design space perfectly; such an assumption is not justifiable in the presence of a small number of initial samples. Therefore, the strategy of using option 2, ${\varvec{V}}^{(i)}$ and ${\varvec{W}}_1^{(i)}$, is preferable.
2. In practical engineering settings, the physical models used for generating training samples could be expensive, therefore, the numbers of samples generated for initial surrogate model construction and in each active learning iteration depend on the available computational resources and staff schedules, which may affect the ultimate improvement achieved. (For example, it may not be practical to try one sample at each iteration, and the analyst may wish to run a batch of samples at each iteration.)
3. As mentioned in Sect. 2.4, if sample-based optimization is used to implement the active learning process, a pool of candidate samples for active variables, ${\mathscr {P}}_{\eta }$ will need to be constructed to start the adaptive improvement process. The proposed active learning strategy considers both exploration (finding samples in the regions where no samples have been proposed) and exploitation (finding samples in regions where the surrogate model performs poorly). The former is affected by the range of the samples in the candidate pool and the latter is affected by how the samples in the pool are distributed (for example, evenly distributed or randomly distributed within a range). As a result, the range and distribution of the samples in the candidate pool will affect the ultimate improvement achieved by the active learning.
4. The parameter $\alpha$ of the active learning function, which balances exploration and exploitation, may affect the adaptive improvement; this is a limitation for learning functions that consider both exploration and exploitation. Depending on the specific application and available computational resources to obtain training samples, a variety of settings for $\alpha$ may be investigated in the adaptive improvement process and the strategy that improves the results most may be decided. For example, if a very limited number of samples is available at the beginning of the active learning process, the analyst may choose to start with $\alpha = 1$, and after a few iterations switch to a hybrid exploration-exploitation strategy, i.e., $\alpha \in (0, 1)$.
5. A subset of features are chosen to build surrogate models, and reconstruction error is incurred by omitting other features. In addition, the residuals in the surrogate models will also contribute to the total error. In the algorithm described in Sect. 2.3, a surrogate model is first constructed in the active subspace that is calculated using all samples in the training set ${\mathscr {D}}$; we denote the mapping between this active subspace and the original input space as ${\varvec{W}}_1^{{\mathscr {D}}}$. The mapping between the subspace defined by the samples in the testing set ${\mathscr {D}}_{t}$ and the original input space is denoted as ${\varvec{W}}_1^{{\mathscr {D}}t}$. When testing the performance of the surrogate, the samples in the testing set ${\mathscr {D}}_{t}$ are mapped to the subspace defined by ${\varvec{W}}_1^{{\mathscr {D}}}$. However, the samples in both sets are in fact in different subspaces (${\varvec{W}}_1^{{\mathscr {D}}}$, ${\varvec{W}}_1^{{\mathscr {D}}t}$), which are different from an unknown true subspace of the problem; in testing the surrogate model, the error caused by the orientation difference between the two subspaces also contributes to the total prediction error. One way to quantify the difference in the orientations between the two high-dimensional vectors ${\varvec{W}}_1^{{\mathscr {D}}}$ and ${\varvec{W}}_1^{{\mathscr {D}}t}$ is to calculate the cosine of the angle between the two vectors, which is given by the dot product:
  5 $$\begin{aligned} cos<{\varvec{W}}_1^{{\mathscr {D}}},\ {\varvec{W}}_1^{{\mathscr {D}}t}>\ =\ \frac{{{\varvec{W}}_1^{{\mathscr {D}}} \cdot {\varvec{W}}_1^{{\mathscr {D}}t}}}{{\Vert {\varvec{W}}_1^{{\mathscr {D}}} \Vert \cdot \Vert {\varvec{W}}_1^{{\mathscr {D}}t}\Vert }} \end{aligned}$$

If the two orientations ${\varvec{W}}_1^{{\mathscr {D}}}$ and ${\varvec{W}}_1^{{\mathscr {D}}t}$ are the same, the angle between them is zero and the cosine value in Eq. (5) is 1.

Thus, multiple sources of errors (reconstruction error, surrogate error, and orientation error) contribute to the overall error of the surrogate prediction. The relative contributions of these different errors should be considered in evaluating the performance of the adaptive learning process. Some of these errors may not be reducible for a given application problem; thus there may be a lower limit beyond which the surrogate prediction could not be improved. This point will be illustrated in the next section.

## Evaluation of the proposed approach using benchmark problems

Our proposed methodology aims to improve the surrogate models in the low-dimensional space, and then mapping the surrogate prediction back to the original high-dimensional space. Therefore the adaptive learning process happens in the low-dimensional space and the surrogate models we want to improve are indeed low-dimensional. The strategy to improve these surrogate models is to build a learning function that considers both exploration (based on a distance measure) and exploitation (based on surrogate error approximations). In this section, the proposed active learning strategy is tested using three benchmark test functions listed in the “Appendix”. The benchmark test functions $f_b({\varvec{x}})$ are of different complexity, each with a two-dimensional input and a single output. Based on Jones et al. (1998), the initial design should include 10*n* samples where *n* is the dimension of the input space. Thus, for each benchmark test functions, 20 settings of ${\varvec{x}}$ are generated using the maxmin Latin hypercube sampling (Stein 1987; Morris and Mitchell 1995; Jin et al. 2003) approach. These samples of ${\varvec{x}}$ are evaluated in $f_b({\varvec{x}})$ to formulate an initial training set ${\mathscr {D}}_{0, b}$ for initial surrogate model construction. A GP regression surrogate model $\hat{G}_b({\varvec{x}})$ is built for each test function $f_b({\varvec{x}})$. Considering the dimension and computational cost, the proposed active learning function in Eq. (4) takes the form:

6 $$\begin{aligned} {\mathcalligra{l}}_b({\varvec{x}})\,= \,& {} \alpha \times \frac{\Vert {\varvec{x}}-{\varvec{x}}^{\text {train}}\Vert }{N \cdot \max _{p \ne q} \Vert {\varvec{x}}_p^{\text {train}} - {\varvec{x}}_q^{\text {train}}\Vert }\nonumber \\{} & {} +(1-\alpha ) \times |\hat{G}_b({\varvec{x}}) - f_b({\varvec{x}})| \end{aligned}$$

Note that this learning function is a simplified form of Eq. (4), because there is no high-dimensional input or output, and there is no space mapping. However, it balances exploration (based on distance measure) and exploitation (based on surrogate error), and thus captures the core components in Eq. (4). Given a candidate pool ${\mathscr {P}}_b$, the maximizer of the learning function, ${\varvec{x}}^*$, which can be expressed as

$$\begin{aligned} \begin{aligned}&\underset{{\varvec{x}}}{\text {argmax}}{} & {} {\mathcalligra{l}}_b({\varvec{x}}) \\&\text {subject to}{} & {} {\varvec{x}} \in {\mathscr {P}}_{b}, \end{aligned} \end{aligned}$$

is chosen as the input of the new training sample at each iteration. The training set is updated as ${\mathscr {D}}_i = {\mathscr {D}}_{i - 1} \bigcup {({\varvec{x}}^*, f_b({\varvec{x}})})$, where *i* is the index number. At each iteration, the surrogate model is evaluated on a separate set of 20 testing samples, using the normalized root mean squared error (NRMSE) metric, which is defined as:

7 $$\begin{aligned} NRMSE = \frac{\sqrt{\Sigma _{i = 1}^{20}(f_{b, i} - \hat{G}_{b, i})/n}}{f_{b, {\text{max}}} - f_{b, {\text{min}}}} \end{aligned}$$

**Fig. 2:**
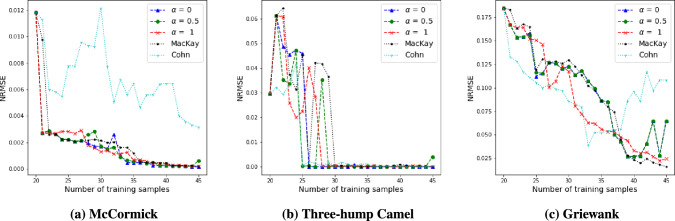
NRMSE vs. number of training samples for three benchmark test functions

For each benchmark test function, $\alpha$ = 0, 0.5, and 1 are used and in each case, we repeat the adaptive iterations for 25 times. The plots of surrogate model accuracy (in terms of NRMSE) versus the number of training samples are shown in Fig. 2.

For the Griewank function, strategies with $\alpha$ = 0 and 0.5 do not come to a steady improvement toward the end, however, it can be observed that the accuracy of the surrogate models are improved by adding more samples that are selected using the active learning function [Eq. (6)] for all cases. The three benchmark test functions are of different complexity (plate-shaped, valley-shaped, and containing many local minima, respectively, as shown in “Appendix”). They have two-dimensional input and one-dimensional output. The learning function for the benchmark test functions is a simplified version of the one for the high-dimensional problem. It contains an exploration term (based on distance measure) and an exploitation term (based on surrogate error).

For all three test functions, we compared our approach against the active learning strategies proposed by MacKay (1992) and Cohn (1993). In MacKay’s approach, the aim is to maximize the expected information gain regarding the parameters of the model; this is achieved by selecting the new training point where the surrogate model output has the maximum variance. In Cohn’s approach, the goal is to minimize the generalization error; this is achieved by selecting the training point which minimizes the mean-square error (MSE) of the estimator. MSE is first decomposed into a variance term and a bias term and the contribution of bias is assumed to be small compared to the variance contribution. Therefore, the method estimates the change of variance of the surrogate model output for a candidate point with respect to a reference point, and the change of variance is averaged over all the previous training points. Thus both these methods use criteria based on variance to select new training points, whereas our proposed method uses the bias in the exploitation term. This choice is based on Hombal and Mahadevan (2011) where it is shown that using the bias captures nonlinearities in the underlying function more effectively than using the variance as the criterion.

It is shown in Fig. 2 that for all three benchmark problems, our method has similar performance as MacKay’s approach, and for two out of three problems, our method has better performance than Cohn’s approach. Note that the choice of GP surrogate model is purely for demonstration purposes, and any appropriate surrogate model form could also employ with the proposed active learning strategy.

In the next section, we demonstrate the active learning strategy for an engineering example, in predicting high-dimensional quantities related to the additive manufacturing part.

## Engineering application

### Problem description

Electron beam melting (EBM) is an additive manufacturing (AM) process of fusing powder particles, layer-upon-layer, using an electron beam as the energy source. The process is typically used in the case of metals and metal alloys. Multiple passes of a low power electron beam are used for heating and sintering the powder bed prior to selective melting. For the application problem in this study, we focus on the thermo-mechanical behavior of an AM part produced by the EBM process. For this purpose, we have developed a finite element-based thermal analysis model to simulate the thermal response of the part and a finite element-based mechanical model that uses the thermal response to estimate the residual stress at the end of the cooling phase. Note that the stress is computed at the end of a single pass of the electron beam. In this study, the thermal and mechanical models are weakly coupled (Debroy et al. 2017), i.e., the temperature distribution is used as an input heat load for the mechanical model. Finite element analysis is performed using Abaqus. Details of the two models can be found in Vohra et al. (2020) and are not discussed here, for the sake of brevity.

**Fig. 3:**
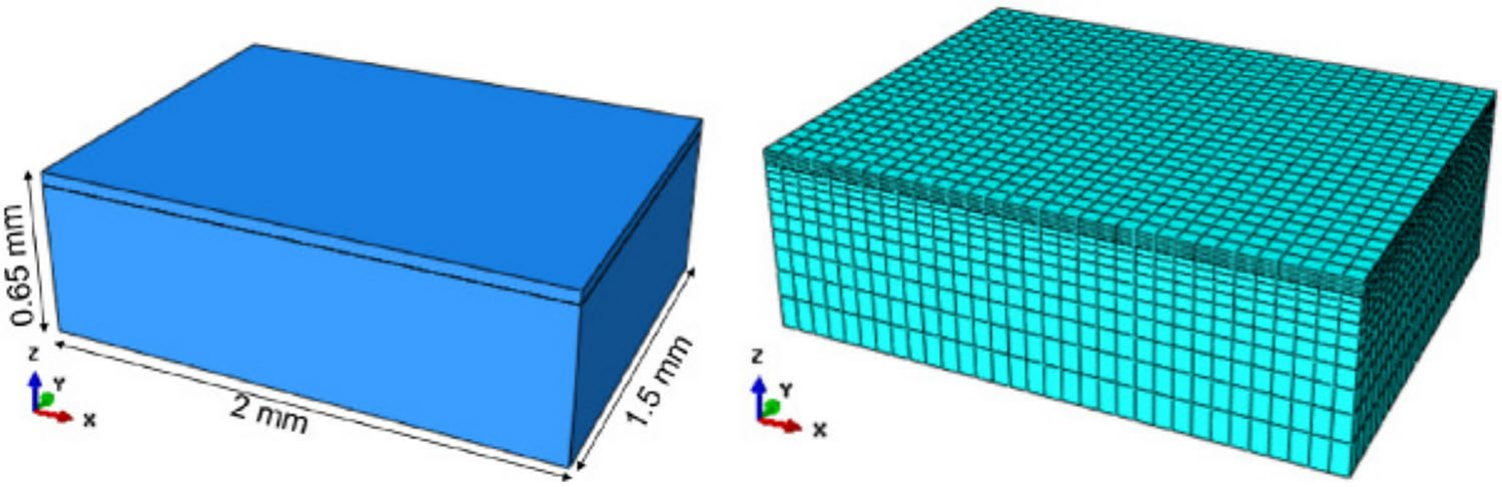
Part geometry and the corresponding mesh as modeled in Abaqus (Vohra et al. 2020)

The output of interest is the residual stress in the part as a result of a single scan of the electron beam along its length. A part of dimensions (in mm), 2 $\times$ 1.5 $\times$ 0.65, with layer thickness of $50\,\upmu \text {m}$, is built as shown in Fig. 3 (left). The process of laying the new powder on bulk material formed by previous scans is simulated by activating the initially deactivated elements representing the powder layer. To mitigate the computational cost associated with FEA, a non-uniform mesh is employed wherein a finer mesh is considered for the powder region where the heat flux is applied. A gradually coarsening mesh is considered for the bulk material, significantly far from the heat source as shown in Fig. 3 (right). The mesh consists of 13,200 nodes and 10,752 elements in total. The material used to manufacture the part is Ti6Al4V and its thermophysical properties considered in the finite element analysis are provided in Table 1.

**Table 1:** Thermophysical properties of Ti-6Al-4V

Density $\left( \textrm{kg} / \textrm{m}^3\right)$	4428
Solidus Temperature $\left( { }^{\circ } \textrm{C}\right)$	1605
Liquidus Temperature $\left( { }^{\circ } \textrm{C}\right)$	1655
Latent heat $(\textrm{J} / \textrm{kg})$	365,000
Elastic Modulus (GPa)	110
Poisson’s ratio	0.41
Yield strength (MPa)	825

Simulations are performed on a workstation with a system configuration Intel Core i7-4790 CPU, 3.60 GHz with 16GB RAM. It is observed that on average the thermal model takes 20 min, and the mechanical model takes 10 min to complete the simulation pertaining to a single pass of the electron beam. Note, however, that the simulation duration depends on the choice of values for the set of inputs. Moreover, a weak coupling assumption leads to a computational time of 30 min to generate 1 training point for the surrogate model. On the other hand, a strong coupling assumption would take about 150 min (considering an average of 5 iterations needed for convergence between the thermal and mechanical analyses) to generate 1 training sample. A weak coupling assumption is used here; it does introduce approximation errors, however, it has been shown to capture experimental trends with reasonable accuracy in similar applications (Farahmand and Kovacevic 2014). The von Mises stress at the end of the cooling process is considered as the indicator of residual stress in the AM part (Vastola et al. 2016) and used as QoI.

### Surrogate model construction and evaluation

A set of surrogate models (*one for each dominant feature*, as explained in Sect. 2.3) is constructed for the residual stress field, ${\varvec{S}}$ at the cross-section of the part ($x-z$ plane in Fig. 3) passing through its center. We will refer to this plane as $x^c-z^c$ in the remainder of this paper. The surrogate model maps three sets of inputs, namely, the process parameters ($\varvec{\theta }_P$), mechanical properties ($\varvec{\theta }_M$), and thermal properties ($\varvec{\theta }_T$) to the output stress field. Note that the surrogate model maps a deterministic set of input values (parameter values) to a deterministic output (stress field).

The set of process parameters includes beam power (*P*), scan speed (*v*), and pre-heat temperature ($T_0$). Mechanical properties include yield strength (*Y*), elastic modulus (*E*), and bulk density ($\rho$). Thermal properties include specific heat ($C_p$) and bulk thermal conductivity ($\kappa$). Note that $C_p$ and $\kappa$ are considered to be functions of the local temperature, *T*. Specifically, a polynomial of degree 2 is fit to a set of data pertaining to the variation of $C_p$ and $\kappa$ with temperature (20–1655K), as provided in Fu and Guo (2014). Hence, a total of 12 parameters ($\varvec{\theta }$) are mapped to the stress field including coefficients of the polynomial fits corresponding to $C_p$ and $\kappa$. A uniform probability distribution in a range: $[0.9\varvec{\theta }_0, 1.1\varvec{\theta }_0]$, where $\varvec{\theta }_0$ denotes a vector of nominal values, is considered for each parameter. Nominal values of the mechanical properties: *Y*, *E*, and $\rho$ are provided in Table 1. Nominal values of the process parameters and temperature coefficients for the thermal properties are provided in Table 2. It must be noted that the choice of a uniform probability distribution for $\varvec{\theta }$ indicates that any value in the considered range for a given parameter has a probability value of $1/(u - l)$ (*u*: upper limit, *l*: lower limit) associated with it. Predictions of the original physics model at a collection of training samples, generated using OSLH in the input space is used to train the surrogate model for each feature ${\mathscr {L}}_j$ as discussed further below.

**Fig. 4:**
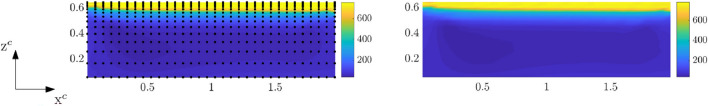
Residual stress field in the $x^c-z^c$ plane (Vohra et al. 2020)

The output quantity of interest here is stress, which is computed on a 2-dimensional non-uniform grid comprising 32 points along the length ($x^c$) and 14 points along the height ($z^c$) as highlighted in Fig. 4 (left). By considering stresses in all 6 directions, the dimension of ${\varvec{S}}$ is 2688. The mesh size was selected such that a converged solution was obtained within a reasonable amount of computational effort. A finer mesh is used near the part surface since sharp thermal gradients lead to a larger amount of stress in this region, as shown in Fig. 4.

**Table 2:** EBM process parameters and temperature coefficients for $C_p(C_{i,C_p})$ and $\kappa (C_{i,\kappa })$

Scan speed, *v*(*mm*/*s*)	500
Beam power, *P*(*W*)	160
Pre-heat temperature, $T_0 \left( { }^{\circ } \textrm{C}\right)$	650
Specific heat, $C_p = C_{0,C_p}+C_{1,C_p}T+C_{2,C_p}T^2 (J/kg/K)$	$540(C_{0,C_p}),\ 0.43(C_{1,C_p}),\ -3.2\times 10^{-5}(C_{2,C_p})$
Thermal conductivity, $\kappa = C_{0,\kappa }+C_{1,\kappa }T+C_{2,\kappa }T^2 (W/m/K)$	$7.2(C_{0,\kappa }),\ 0.011(C_{1,\kappa }),\ 1.4\times 10^{-6}(C_{2,\kappa })$

#### Generation of initial training samples and testing samples

As stated above, an initial set of training samples ${\mathscr {D}}_0$ and testing samples ${\mathscr {D}}_t$ are generated separately using OSLH; the number of samples in these sets are denoted as $N_0 = card\{{\mathscr {D}}_0\}$ and $N_t = card\{{\mathscr {D}}_t\}$. Note that both sets contain data obtained from the expensive thermal and the mechanical models. The residual stress field is initially computed on the $x^c-z^c$ plane for $N_0$ pseudorandom samples in the 12-dimensional input domain.

#### Dimension reduction

In order to address the challenge of high dimensionality in both the input and output spaces, the surrogate models are constructed for low-dimensional representations of both the inputs and outputs. Dimension reduction is performed as described in Sect. 2, using SVD for the outputs and active subspace discovery for the inputs. In this numerical example, the number of features to build the surrogate models, $K^*$, is set to three, since the top three features account for 96.36%, 2.45%, and 0.91% of the variance in the output QoI, respectively. For each feature, ${\mathscr {L}}_j$ for $j = 1, 2, 3$, the corresponding active subspace is then computed. The number of active variables is set to one for all the features because the variance explained by the first eigenvector, i.e., the first column of ${\varvec{W}}$, is over 95% for all three features. We inspected the product of the active space mapping vectors of any two features and found that ${\varvec{W}}_{1(i)}^{\top }{\varvec{W}}_{1(j)} \ne 0$ for $i \ne j,\ i, j = 1,2,3.$ Note that although at each iteration of adaptive surrogate improvement, these percentages will be slightly different given the current number of training samples, they are always greater than 95%. In this example, for the top three features, a linear trend is observed when plotting all the training samples in the corresponding active subspaces, therefore, a linear regression model is used as the form of the surrogate model. Note that the linear regression model is adequate for this example; other appropriate forms of surrogate model could be chosen for other application problems.

We also repeated the above analyses using four and five features, as well as using a larger number of active variables; no significant improvement was observed.

#### Surrogate models and performance metric

The map from $\varvec{\theta }$ to each feature ${\mathscr {L}}_j$ is approximated by a surrogate model ${\mathscr {L}}_j(\varvec{\theta }) = \hat{G}_j(\varvec{\eta }_j)$ using Algorithm 1 in Sect. 2. The output QoI, ${\varvec{S}}$, is reconstructed using surrogate prediction for each feature ${\mathscr {L}}_j$; the reconstructed stress field is denoted using $\hat{{\varvec{S}}}$. We denote the surrogate model constructed at the *i*-th iteration as $\hat{G}^i_j$, and $i=0$ is for the initial surrogate model constructed using ${\mathscr {D}}_0$. At each iteration, the accuracy of surrogate model will be evaluated for each testing sample using the metric:

8 $$\begin{aligned} \epsilon = \frac{1}{N_t}\Sigma_{m=1}^{N_t}\frac{\Vert {\varvec{S}}_m - \hat{{\varvec{S}}}_m\Vert }{\Vert {\varvec{S}}_m\Vert } \end{aligned}$$

which is the average of the relative *l*2-norm of the difference in the prediction of the residual stress field.

### Investigation of issues affecting adaptive improvement of the surrogate model

As discussed in Sect. 2.5, there are several issues affecting the active learning process, including the number of samples, the range and distribution of the adaptive training samples, contributions of various errors, and the importance of exploration versus exploitation in the learning function. These issues are investigated with the numerical example in this subsection. Note that in this numerical example, for a new candidate training sample for adaptive surrogate improvement, the exploitation term in the active learning function is computed using the surrogate error at the existing training sample that is closest to the candidate training sample. Other approaches to estimate the surrogate error at the candidate training sample are also possible, as discussed earlier.

#### Number of training samples

We first investigate the influences of both the size of the initial training set and the number of newly added training points in each iteration. Note that in this investigation, other issues, i.e., range and distribution of points in the candidate pool, and weighting parameter $\alpha$ in the learning function are kept the same for all trials.

To study the size of the initial training set, we choose to generate $N_0 =$ 13, 20, and 40; the size of the testing set $N_t$ is 20. Note that the original input dimension is 12, therefore, the minimum size of the initial training set should be 13 considering the gradient calculation for active subspace calculation using the algorithm listed in Vohra and Mahadevan (2019). The sizes 13, 20, and 40 are chosen for illustration purposes. The accuracy of the initial surrogate model in active subspace is evaluated using the original space error metric, $\epsilon$, as explained in Eq. (8). The results are shown in Table 3, and indicate error reduction with increasing number of initial training samples.

**Table 3:** Surrogate model accuracy for different $N_0$

Initial training set size $N_0 = card\{{\mathscr {D}}_0\}$	Original space error $\epsilon$ (%)
13	16.35
20	8.91
40	8.15

**Fig. 5:**
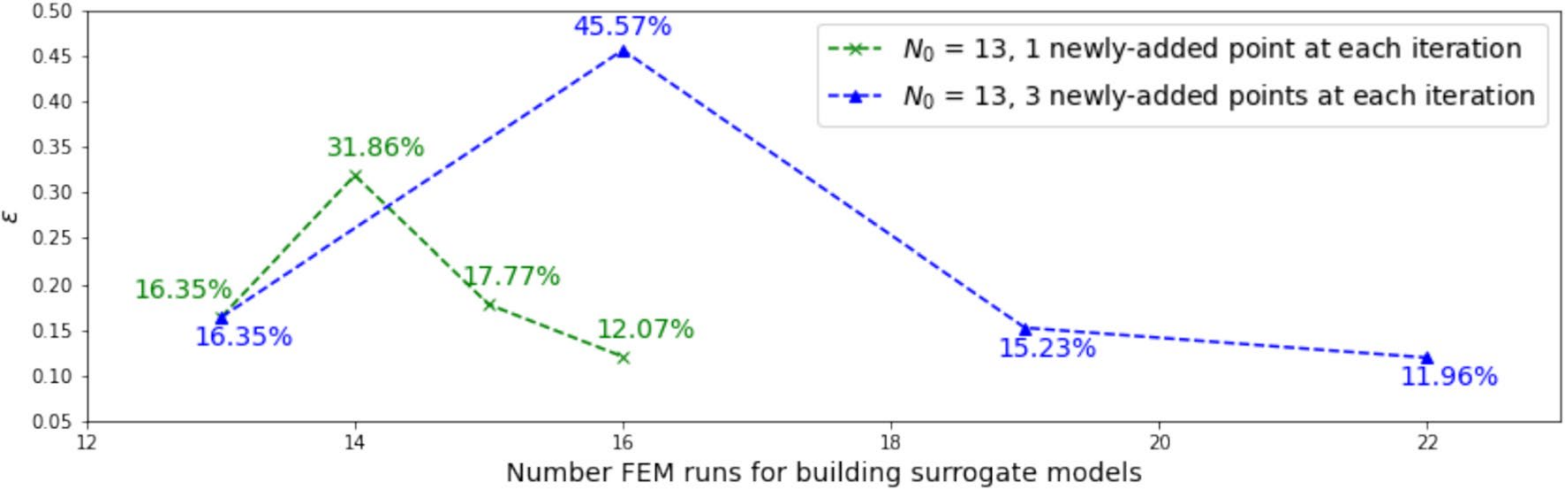
Influence of the number of newly added samples at each iteration, for $N_0$ = 13

**Fig. 6:**
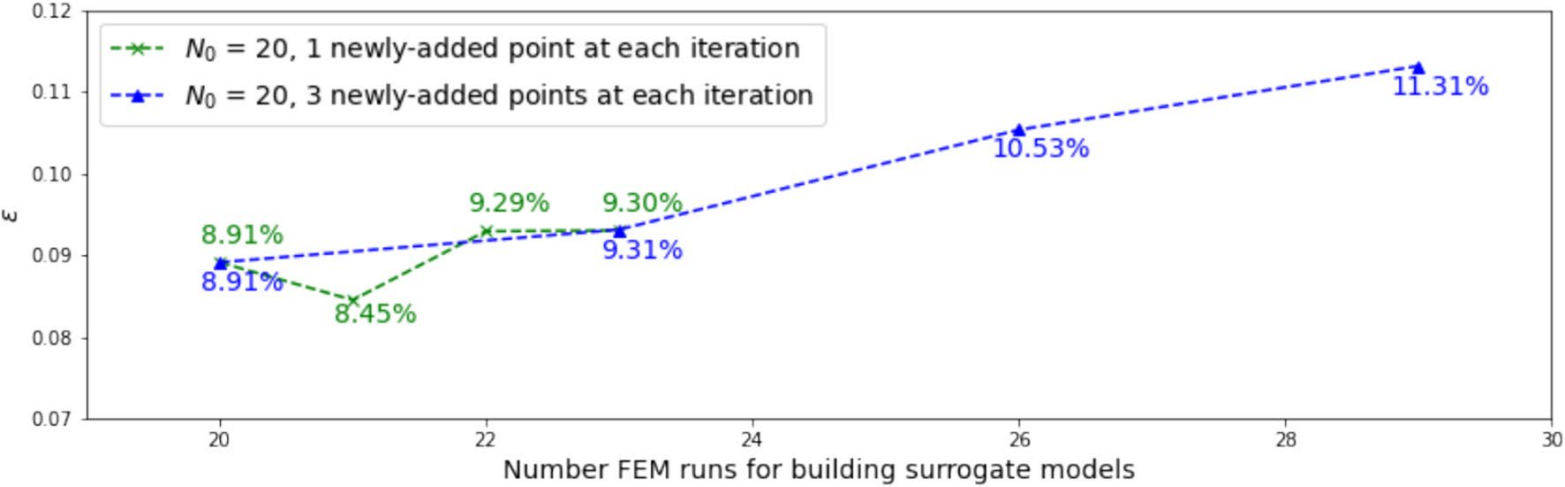
Influence of the number of newly added samples at each iteration, for $N_0$ = 20

To study the influence of the number of newly added samples at each iteration, we experimented with adding one or three new samples at each iteration. With the size of the initial training sets $N_0 = 13$ and $N_0 = 20$, the results are shown in Figs. 5 and 6, respectively. Note that this experiment is conducted by setting the parameter $\alpha$ in the learning function to be 1; results with $\alpha$ = 0 and 0.5 are similar. It is seen that the difference between the accuracies of the surrogate models when adding one point at each iteration and when adding three points at each iteration is small for the $N_0$ = 13 case; for the $N_0$ = 20 case, adding three samples at each iteration results in a larger error after three iterations than adding just one sample each time. In some practical engineering settings, it might be more efficient to add new samples in batches in view of personnel and computing resource availability and scheduling logistics.

#### Range and distribution of samples in the candidate pool

**Fig. 7:**
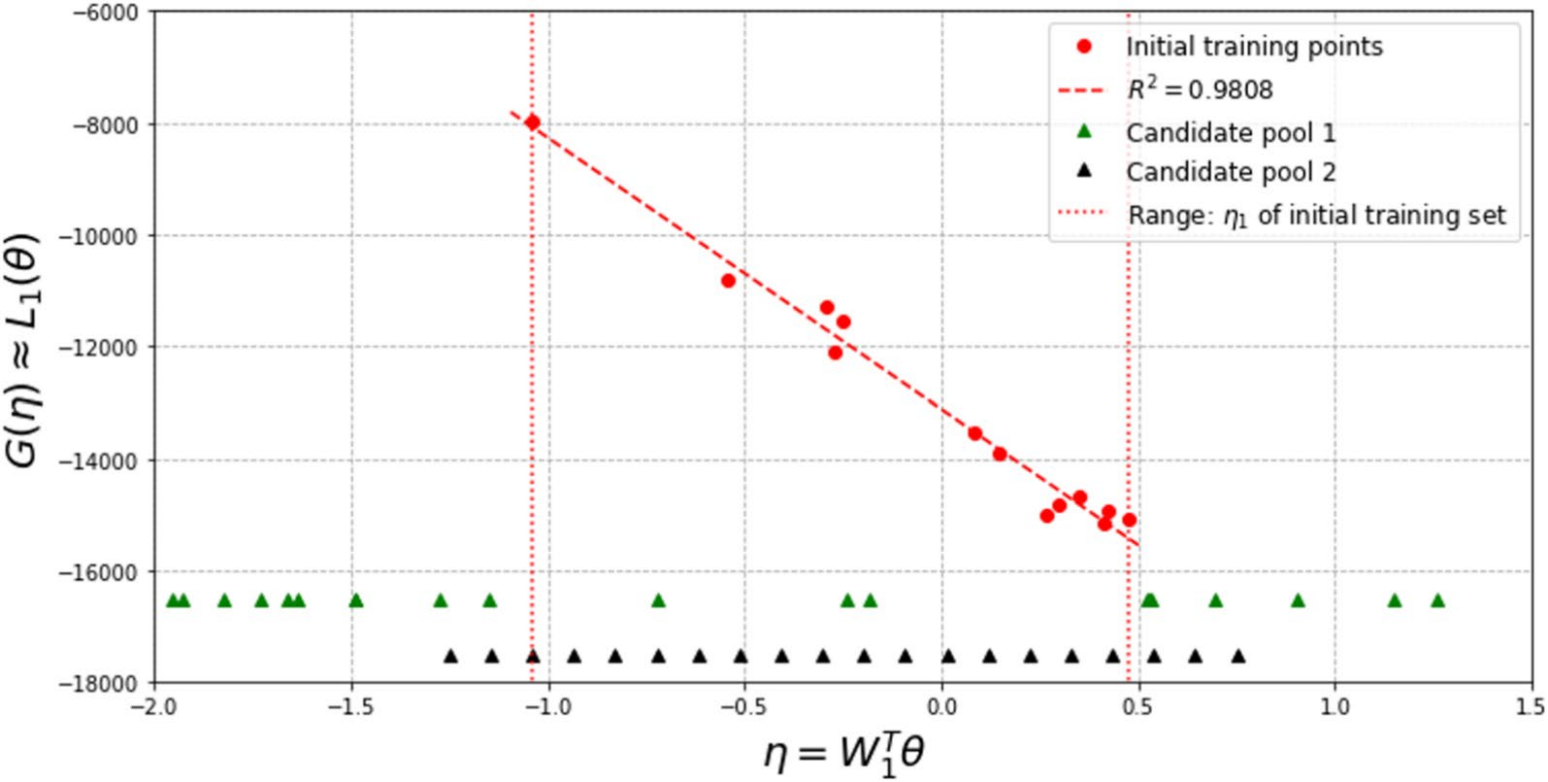
Two candidate pools in active subspace, for $N_0$ = 13

In this investigation, we start with both $N_0 = 13$ and $N_0 = 20$ samples. Dimension reduction is first performed for both the output and the associated input, and the range of the resulting active variable values ${\mathscr {R}}_{N_0}$ are obtained accordingly. The vertical dotted lines in Figs. 7 and 9 indicate the ranges for $N_0 = 13$ and $N_0 = 20,$ respectively.

For $N_0 = 13$, two candidate pools, ${\mathscr {P}}_{\eta }^{(1)}$ (much wider than ${\mathscr {R}}_{N_0}$, with candidates randomly distributed) and ${\mathscr {P}}_{\eta }^{(2)}$ (slightly wider than the ${\mathscr {R}}_{N_0}$, candidates evenly distributed) are considered. ${\mathscr {P}}_{\eta }^{(1)}$ and ${\mathscr {P}}_{\eta }^{(2)}$ are colored in green and black, respectively, in Fig. 7. The red dots in Fig. 7 indicate lower-dimensional representations of the initial training samples, with the y-coordinate indicating the value of the first principal feature (${\mathscr {L}}_1$) and the x-coordinate indicating the active variable values. The red dash line is the surrogate model of feature 1 trained by $N_0 = 13$ samples and the vertical red dotted lines indicate the range of the active variable values for those initial samples.

**Fig. 8:**
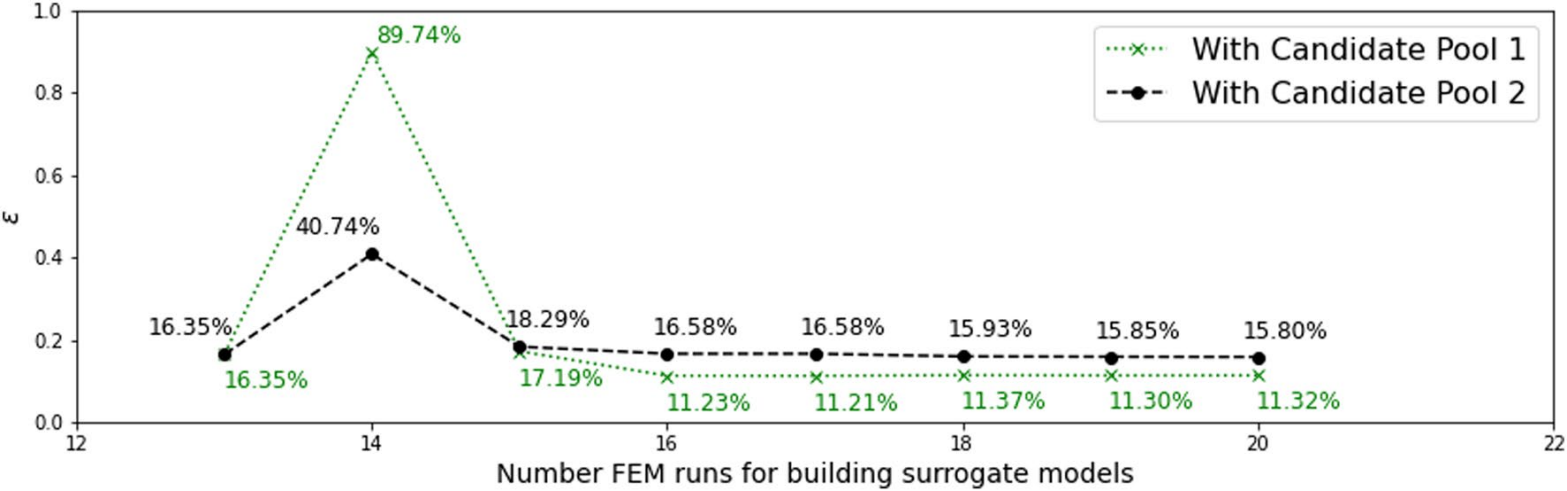
Comparison of active learning with different candidate pools, for $N_0$ = 13

Experiments of adaptive improvement of the surrogate models by optimizing the learning function using both candidate pools are conducted. In each iteration, only one new sample is selected and added to the training set ${\mathscr {D}}$. The results of experiments are compared in Fig. 8. It is observed that the active learning with the wider candidate pool, ${\mathscr {P}}_{\eta }^{(1)}$ resulted in lower error in the end. Note that this experiment is conducted by setting the parameter $\alpha$ in the learning function to be 0. Results with $\alpha$ = 0.5 and 1 are similar to the results with $\alpha$ = 0.

**Fig. 9:**
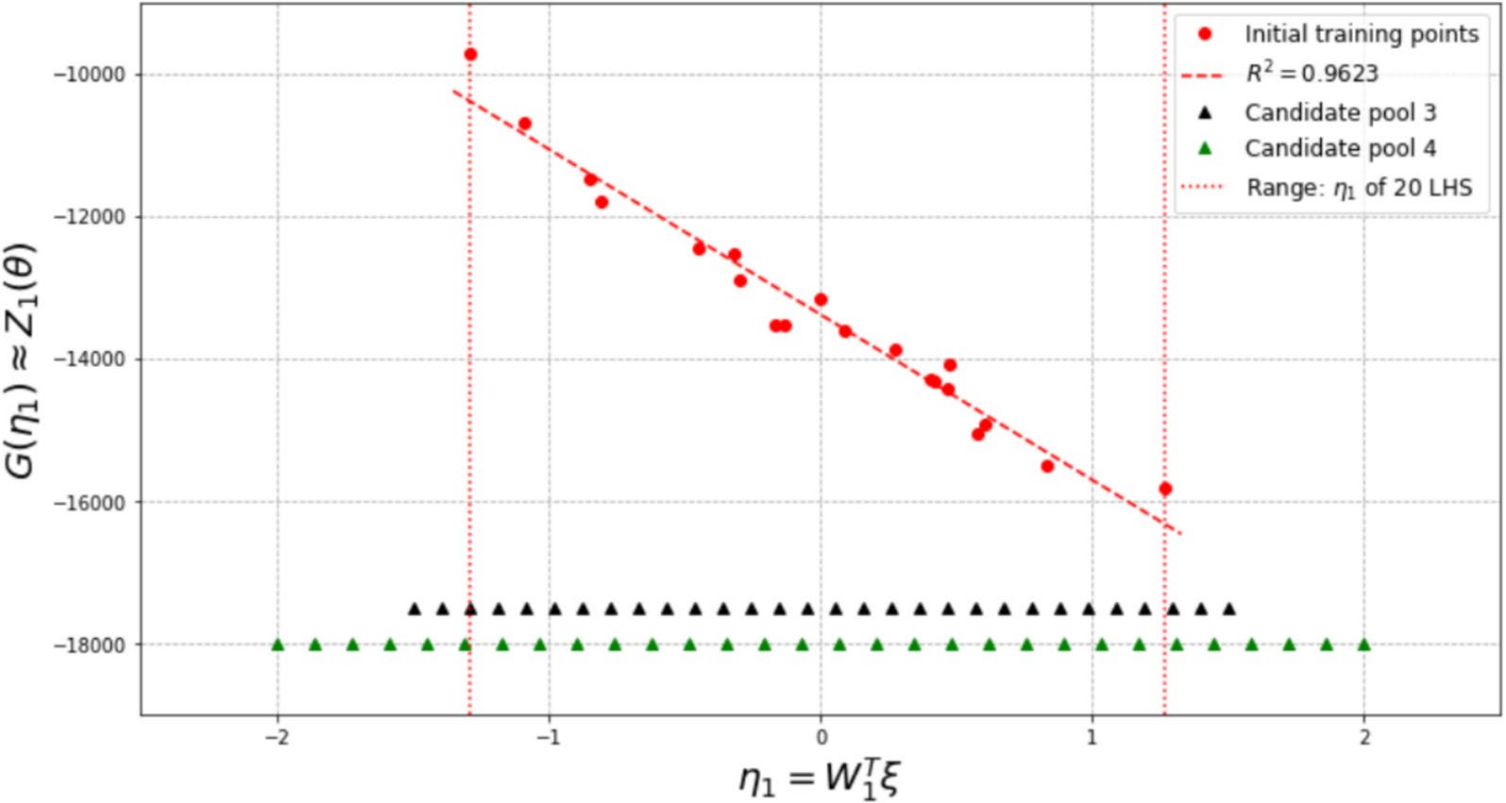
Two candidate pools in active subspace, for $N_0$ = 20

**Fig. 10:**
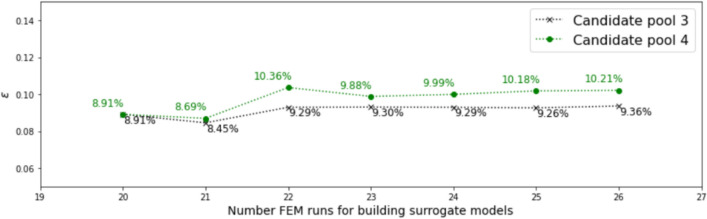
Comparison of active learning with different candidate pool, for $N_0$ = 20

For $N_0 = 20$, two different candidate pools ${\mathscr {P}}_{\eta }^{(3)}$ (much wider than the ${\mathscr {R}}_{N_0}$, candidates evenly distributed) and ${\mathscr {P}}_{\eta }^{(4)}$ (slightly wider than the ${\mathscr {R}}_{N_0}$, candidates evenly distributed) are also generated, as shown in Fig. 9. Experiments of adaptive improvement of the surrogate models using both candidate pools are conducted. In each iteration, only one new sample is selected from the candidate pool and added to the training set ${\mathscr {D}}$. The results of the experiments are compared in Fig. 10. In this case, active learning with the narrower candidate pool, ${\mathscr {P}}_{\eta }^{(3)}$ resulted in lower error in the end. Note that the above experiments in this subsection are conducted by setting the parameter $\alpha$ in the learning function to be 1. Results with $\alpha$ = 0 and 0.5 are similar to the results with $\alpha$ = 1.

**Fig. 11:**
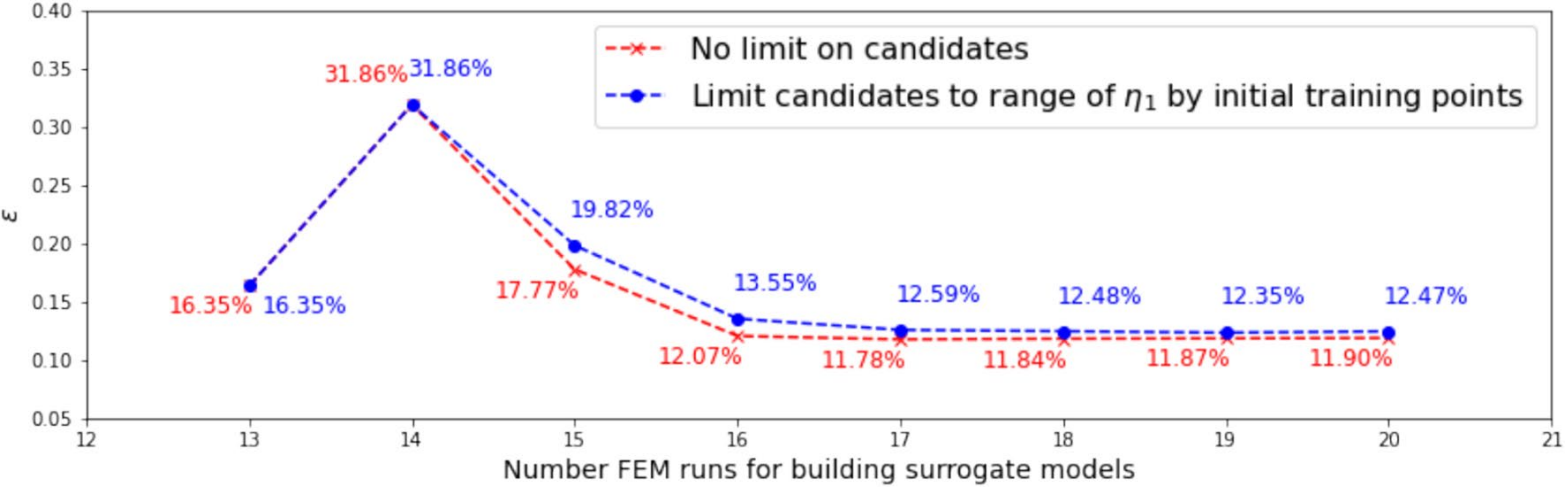
Effect of limiting candidate selection, for $N_0$ = 13

**Fig. 12:**
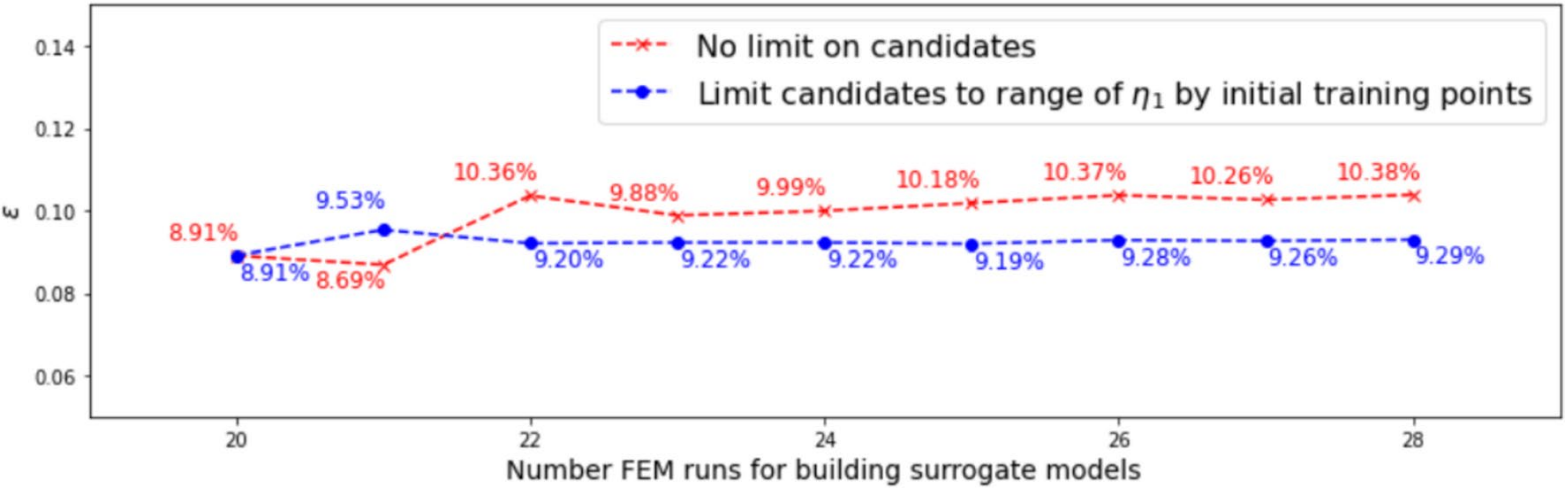
Effect of limiting candidate selection, for $N_0$ = 20

Another set of experiments is also conducted to consider the effects of limiting the candidate point selections within the range of $\eta _1$ decided by the initial set of training points, *i*.*e*., only points within the vertical dotted lines can be chosen and added to the training set at any iteration. In this set of experiments, Fig. 11 shows the results for $N_0$ = 13, candidate pool ${\mathscr {P}}_{\eta }^{(2)}$ and $\alpha$ = 1; and Fig. 12 shows the results for $N_0$ = 20, candidate pool ${\mathscr {P}}_{\eta }^{(4)}$ and $\alpha$ = 0.5. It can be seen that limiting the selection to within the active variable range of the initial training points results in a smaller final error in this example for $N_0$ = 20 initial samples.

In summary, the initial samples play a significant role w.r.t. how the range and distribution of the samples in the candidate pool affect the adaptive improvement process.

#### Weighting parameter $\alpha$ in the learning function

**Fig. 13:**
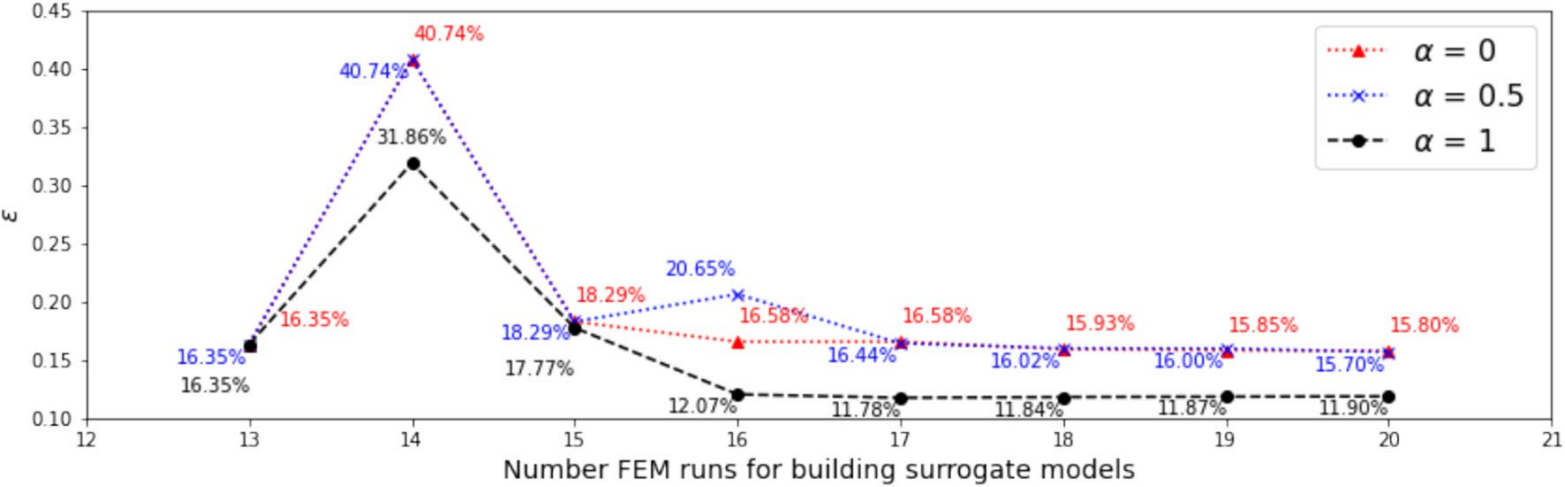
Adaptive improvement with different $\alpha$ values, for $N_0 = 13$

In the active learning function defined in Eq. (4), the parameter $\alpha$ balances exploration and exploitation and affects the active learning process in terms of training sample selection and the improvement of the surrogate. In this experiment, three values of $\alpha$ = 0, 0.5, and 1 are chosen to study its influence. We start with $N_0=13$ samples and the results are shown in Fig. 13. Note that the $\alpha$ = 1 (pure exploration) has the best improvement, indicating that the initial set of 13 training samples ${\mathscr {D}}_0$ does not cover the design space well.

**Fig. 14:**
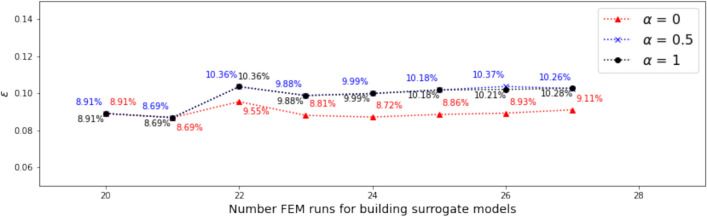
Adaptive improvement with different $\alpha$ values, for $N_0 = 20$

For experiments starting with $N_0 = 20$ samples, $\alpha$ = 0, 0.5, and 1 are chosen. The candidate pool, ${\mathscr {P}}_{\eta }^{(4)}$, in which 30 points are placed in the range [− 2, 2] with uniform spacing, was chosen to conduct this experiment. The testing set ${\mathscr {D}}_{t}$ is a separate set of 20 LHS samples. The results are shown in Fig. 14 and the final error is more than the error with the initial training samples; this will be discussed in the next subsection. For this case, pure exploitation ($\alpha$ = 0) gave the best performance. Comparing the results for $N_0 = 13$ and $N_0 = 20$, it is seen that the initial samples have a strong effect on the relative performance of exploration vs. exploitation.

#### Error sources: reconstruction error and surrogate error

In the above investigations on the effects of the number of training points in each iteration, range and distribution of points in the candidate pool, and the weight parameter $\alpha$ in the learning function, some experiments have a larger final error than the starting error, such as in Figs. 10, 12, and 14.

**Fig. 15:**
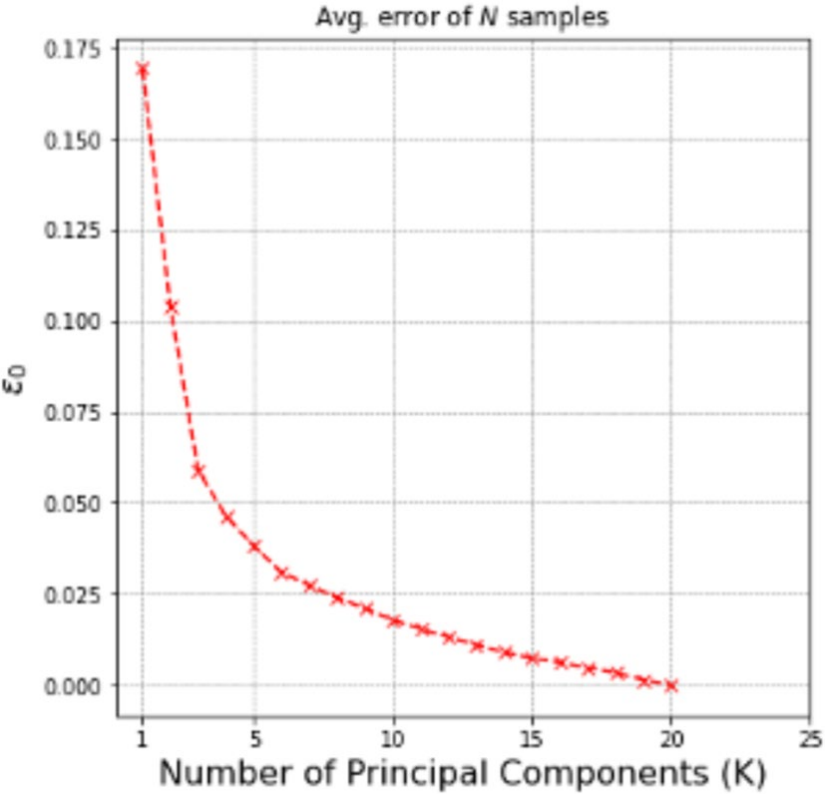
Reconstruction error vs. number of pricipal features

As mentioned in Sect. 4.2.2, based on the amount of variance captured, three principal features of the output were chosen to build the surrogate models. Since only a few principal features of the output are used, reconstruction error is introduced; surrogate models for these features also contribute to the overall error in the prediction of the QoI. In order to reduce the overall error, steps should be taken to minimize both the reconstruction error and the surrogate error. The most effective way to reduce the reconstruction error is to consider more features than just three; also, more accurate surrogate model forms could be chosen to reduce the surrogate error. It is worth noting the reconstruction error level in this numerical example. Taking the experiment started with $N_0$ = 20 samples in the previous section for example, we plot the average prediction error on all testing samples versus the number of principal features used for output QoI reconstruction in Fig. 15. Note that the error shown in the figure does not include any surrogate error. It is observed that the reconstruction error alone with only three features is about 6%, while the overall error in this case is about 8.91% as shown in Fig. 14. As more samples are added, the reconstruction error stays at around 6% without drastic changes. Note that the reconstruction error and the surrogate error are nested (not simply additive) in contributing to the overall error.

**Fig. 16:**
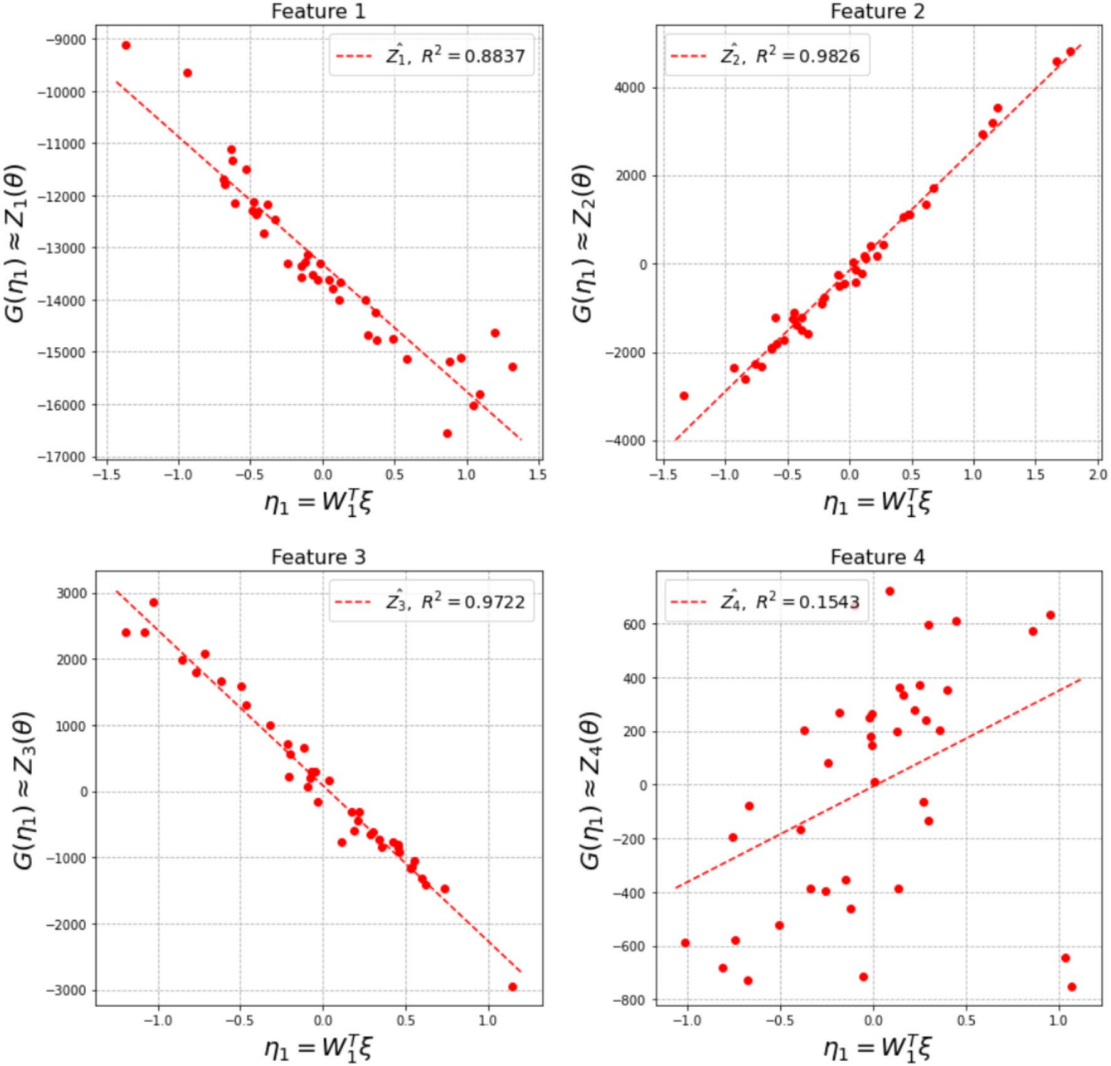
Features 1 to 4 with $N_0 = 40$

In order to reduce reconstruction error and surrogate error, more features as well as more accurate surrogate model forms could be considered. However, in practice, this may or may not improve the surrogate prediction accuracy. For experiments starting with $N_0$ = 40 samples, Fig. 16 shows features 1 to 4, their corresponding first active variables $\eta _1$, and the surrogate model predictions. It can be observed that features 1 through 3 can be well approximated with a linear regression model with just one active variable; for feature 4, a linear regression model in the one-dimensional active subspace is not adequate. We examine whether adding a surrogate model for feature 4 reduces the overall error in the final prediction, and a related issue is the choice of the appropriate model form and the number of inputs for this additional surrogate model.

One option is to consider a higher-dimensional active subspace $(\eta _1,\ \eta _2,\ \eta _3, \ \ldots )$ for constructing the surrogate model of feature 4, *i*.*e*., , approximate feature 4 by a function $\hat{Z}_4(\eta _1,\ \eta _2,\ \eta _3, \ \ldots )$. To examine this option, two active variables $\eta _1$ and $\eta _2$ are considered, and model forms for $\hat{Z}_4$ varying in order and including different interacting terms between $\eta _1$ and $\eta _2$ are conducted. Two of the resulting overall errors in terms of the average error on the testing set in the original space, as defined in Eq. (8), are tabulated in Table 4. The error from using only three features is also listed for comparison.

**Table 4:** Comparison of $\epsilon$ in original space for different surrogate model options with feature 4 with $N_0$ = 40

	$$\epsilon$$ (%)
3 features	8.15
3 features + linear regression surrogate model for feature 4 with one active variable	8.25
3 features + $3\text{rd}$-order surrogate model for feature 4 with two active variables	8.52

Note that $3\text{rd}$ order surrogate model for feature 4 with two active variables listed above is the one with a complete set of interaction terms, *i*.*e*., $\hat{Z}_4(\eta _1,\ \eta _2) = a_0 + a_1\eta _1 + a_2\eta _2 + a_3\eta _1^2 + a_4\eta _2^2 + a_5\eta _1\eta _2 + a_6\eta _1^3 + a_7\eta _2^3 + a_8\eta _1^2\eta _2 + a_9\eta _1\eta _2^2$. Other $2\text{nd}$ order surrogate models with different numbers of interaction terms were also considered; however, all of their $R^2$ values are lower than that of the $3\text{rd}$ order 2D model; therefore, the overall error on the testing set with these surrogate models is larger than 8.52%. From Table 4, it can be observed that including more features and using more complex forms of surrogate models may not reduce the overall error. Similar experiments were conducted with feature 5 but no improvement was observed compared to the three-feature option.

In summary, considering additional features or higher-order surrogate models did not improve the prediction accuracy in this example. However, it is possible in other problems that both reconstruction error and surrogate error might get reduced by considering more features and higher-order surrogate models. Another option to reduce the reconstruction error is to use nonlinear dimension reduction methods, such as autoencoder or diffusion maps, however, methods like autoencoder may need a large number of training samples, which may not be affordable for problems with expensive physics computational models.

#### Orientation difference between active subspaces for training and testing samples

An additional issue affects the assessment of surrogate model accuracy. As discussed in Sect. 2.5, when testing the performance of the surrogate, the samples in the testing set ${\mathscr {D}}_t$ are actually mapped to the subspace defined by ${\varvec{W}}_1^{{\mathscr {D}}}$, which is different from ${\varvec{W}}_1^{{\mathscr {D}}_t}$. If the two orientations ${\varvec{W}}_1^{{\mathscr {D}}}$ and ${\varvec{W}}_1^{{\mathscr {D}}t}$ are the same, the angle between them is zero and the cosine value in Eq. (5) is 1.

For the experiment starting with $N_0$ = 40, using candidate pool ${\mathscr {P}}_{\eta }^{(4)}$ and learning function parameter value $\alpha$ = 0.5, this orientation is calculated and tabulated in Table 5. It can be observed that during the active learning process, the difference in orientations between the two mappings slightly decreases initially and then increases for the latter samples; the two orientations still do not line up at the end, which also contributes to the final overall error.

It is difficult to explicitly quantify the error due to orientation difference. In all the above error calculations, for the testing samples, their inputs are mapped to the active subspaces corresponding to the training samples, using ${\varvec{W}}_1^{{\mathscr {D}}}$. Then the feature values are calculated using the surrogate models that are trained in the low-dimensional space corresponding to the training samples; these feature values are finally used to calculate the QoI in the original space. Thus the error due to the orientation error is nested within the overall surrogate error. The orientation difference as shown in Table 5 can be used as an indicator of the orientation error, and can be monitored with the iterations to qualitatively infer whether the orientation error is increasing or decreasing. In the adaptive learning process in this numerical example, the overall surrogate error is reduced, while the error due to orientation difference is first reduced and then increased.

**Table 5:** Orientations of active subspaces between training and testing set samples with $N_0$ = 40

Number of training samples	40	41	42	43	44	45	46	47
$cos<{\varvec{W}}_1^{{\mathscr {D}}},\ {\varvec{W}}_1^{{\mathscr {D}}t}>$	0.9106	0.8927	0.9027	0.9024	0.9072	0.9136	0.9165	0.9167

We also perform two sets of baseline tests, in which either only the high-dimensional input or the high-dimensional output is considered. Both cases start with $N_0$ = 13 samples and use the same testing set as shown in Fig. 8. In the case where only high-dimensional input is considered (baseline case 1), the stress at the center of the manufactured part is predicted as the QoI (scalar output). After seven iterations, the error on the testing set goes from 55.46% to 80.81%. A possible reason for not observing improvement is poor quality of the initial surrogate model. Note that pure exploitation is used in this case ($\alpha$ = 0). When using pure exploitation, the newly selected samples are based on the current surrogate model error, as indicated in the learning function. Starting with only 13 samples, the error of the initial surrogate model is very high (55.46%). It has been seen in previous studies (Bichon et al. 2013) that there is high fluctuation in the early stages of adaptive improvement before the method starts to converge; thus given the poor quality of the initial surrogate, the increase in error over 7 iterations might reflect this early stage. Even in Fig. 8, there is a large fluctuation in error in the beginning. For the case where only high-dimensional output is considered (baseline case 2), for each feature, two inputs are selected for surrogate model construction (i.e., second and fifth variables in the original 12-dimensional input. Note that this selection is for demonstrating the result for managing high-dimensional output only.) After seven iterations, the error on the testing set goes from 17.06% to 17.01%. Compared to the results shown in Fig. 8, the improvement of the surrogate model is not significant. A possible reason for this lack of improvement is that only two inputs were considered, thus the surrogate model is missing the effect of the remaining 10 inputs and therefore the error is not getting reduced with additional samples. In terms of the magnitude of improvement, the proposed method, in which both the input and output dimension reduction are considered, outperforms the baseline cases, in which reduction of only the input dimension or output dimension is considered. In addition, the proposed method does not need to build separate surrogate models for every QoI location (baseline case 1), nor does it need to make additional judgement in selecting a subset of input variables (baseline case 2).

Other forms of surrogate model, such as a feedforward neural network, were also tested. In the neural network, considering both coordinates (*x*, *z*) and the original 12 inputs, the input to the neural network has 14 dimensions. The output of the neural network is the residual stress at node (*x*, *z*). (This is a scalar output, thus no output dimension reduction is necessary). The neural network has two hidden layers with 64 and 32 nodes, respectively. The mean squared error loss function and the Adam optimizer are used. With input–output data from 13 FEM runs, considering all 448 nodes in each FEM analysis, the total number of data points are $13\times 448 = 5824$. 67% are used for training and 33% are used for testing. The training are performed for 200 epochs and the batch size is set to be 10. The $R^2$ scores of the training and testing sets are $4.09 \times 10^{-3}$ and $4.35\times 10^{-3}$. The neural network does not work well for the additive manufacturing case. Although the stresses at all nodes are spatially correlated, they are the results from a complex thermal-mechanical process with the 12 original inputs, and not necessarily a function of the location. By setting the location as input to the neural network for predicting the residual stress, the assumption is that the residual stress is also a function of the location. This assumption does not seem to hold in this example. By increasing the model’s capacity (adding more layers or neurons) or changing its architecture, the neural network might perform better; however, consider the limited number of training samples we have available, a neural network with more layers or neurons is not feasible. However, considering the location as input may be beneficial in other applications, such as damage localization in non-destructive testing using vibration tests (Miele et al. 2023).

Note that nonlinear dimension reduction methods, such as isomap (Balasubramanian and Schwartz 2002), locally linear embedding (Roweis and Saul 2000), Laplacian eigenmap (Belkin and Niyogi 2003), and diffusion map (Coifman et al. 2005; Coifman and Lafon 2006) provide a unique mapping from original to low-dimensional space, in which case our method can be readily used. If a nonlinear dimension reduction method introduces noise [such as a Bayesian autoencoder (Mescheder et al. 2017)], or if the training data are noisy (the noise and the QoIs are mapped into the lower-dimensional space simultaneously by dimension reduction), well-established surrogate modeling techniques in the literature can be used for noisy data, such as for linear regression models (Muthukumar et al. 2020), Gaussian process models (Stegle et al. 2008), etc.

In summary, in this numerical example, residual stress analysis of an additively manufactured part was used to demonstrate the adaptive improvement of the surrogate modelings with high-dimensional input and output. The effects of the following aspects on the active learning process are investigated: the number of training points, the range and distributions of samples in the candidate pool for adaptive learning, and the weighting parameter of exploration versus exploitation in the learning function. Errors from different sources, both explicit errors (including reconstruction error and surrogate error) and implicit error (error due to orientation difference) are examined. The relative contributions of different error sources determine the amount of improvement that can be achieved in surrogate model prediction within the available computational resources.

## Conclusion

Adaptive surrogate modeling is challenging when both input and output are high-dimensional. When the original physics model is expensive to run, only a limited number of training samples can be generated; therefore the accuracy of the surrogate model needs to be adaptively improved with an active learning strategy. In this work, the principal features of the output are first identified and the active subspace methodology is used to reduce the input dimension. The surrogate models for the dominant features are subsequently built within their corresponding active subspaces and an active learning strategy is proposed to improve the surrogate model and adaptively select the training samples of the original, expensive physics model by balancing exploration and exploitation. The learning function is deployed in the low-dimensional space and the newly added training sample can be easily mapped back to the original space for running the physics model. In coordination with dimension reduction in the output, the methodology proposed in this paper has two novel features: the proposed active learning strategy is conducted in a low-dimensional space; the active learning function properly considers the mappings between different subspaces. The proposed method is demonstrated for a multi-physics additive manufacturing component with a high-dimensional output and multiple input variables including process variables and material properties. Investigations of different issues in the proposed method are conducted, including the number of training samples, range and distribution of samples in the candidate pool for adaptive learning, the relative contributions of error sources, and the relative importance of exploration versus exploitation.

The adaptive learning strategy proposed in this work is a‘zero-th order’ approach in the sense that bias (for exploitation) and distance (for exploration) are used. In the future, this strategy can be extended to a ‘higher-order’ method, in which derivatives or gradients can be incorporated in the learning function; this is a challenging issue in the presence of high dimensionality and complex multi-physics simulations. Physics constraints may also be included in the learning function to reduce the gap between the surrogate model and the actual physics.

## Figures and Tables

**Table 6:**
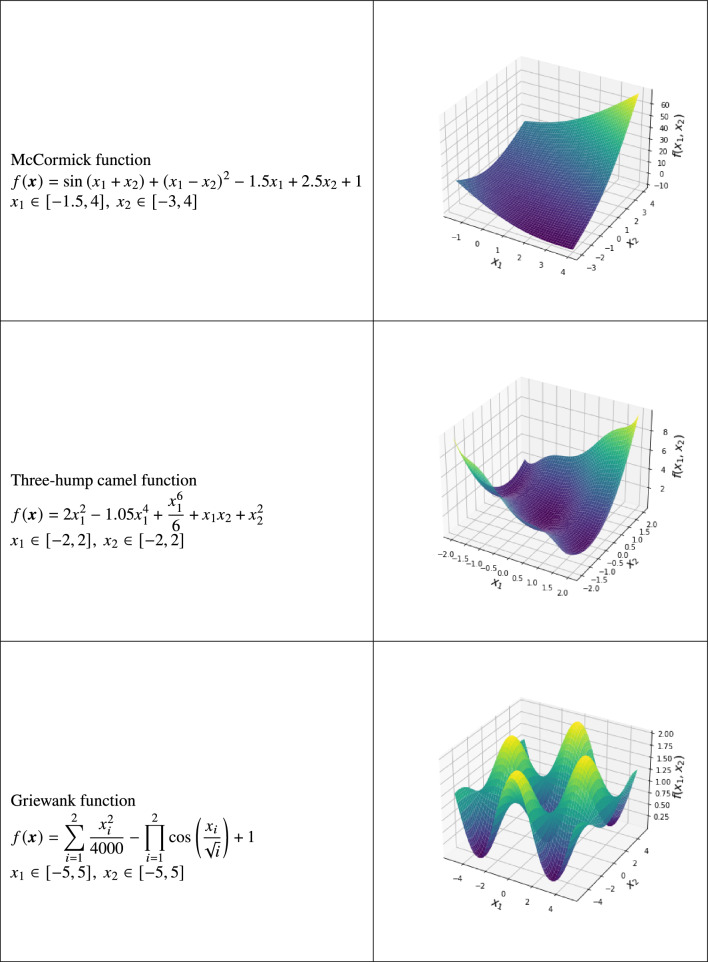
Benchmark test functions

## Appendix

Three benchmark functions with two-dimensional input and one-dimensional output are used to test the efficacy of the proposed active learning method. The functions are defined in Table 6.

## Declarations

### Replication of results

The data used to support the findings of this study are available from the corresponding author upon request.
